# Supplementary material for: In-plane strain engineering in ultrathin noble metal nanosheets boosts the intrinsic electrocatalytic hydrogen evolution activity
Source: Nat Commun. 2022 Jul 20;13:4200. doi: 10.1038/s41467-022-31971-4 (PMC9300738; doi:10.1038/s41467-022-31971-4)
Supplement: Supplementary file 1 — Supplementary Information [file 41467_2022_31971_MOESM1_ESM.pdf]

Supplementary Information

In-plane Strain Engineering in Ultrathin Noble Metal Nanosheets Boosts the Intrinsic Electrocatalytic Hydrogen Evolution Activity

Wu *et al*.

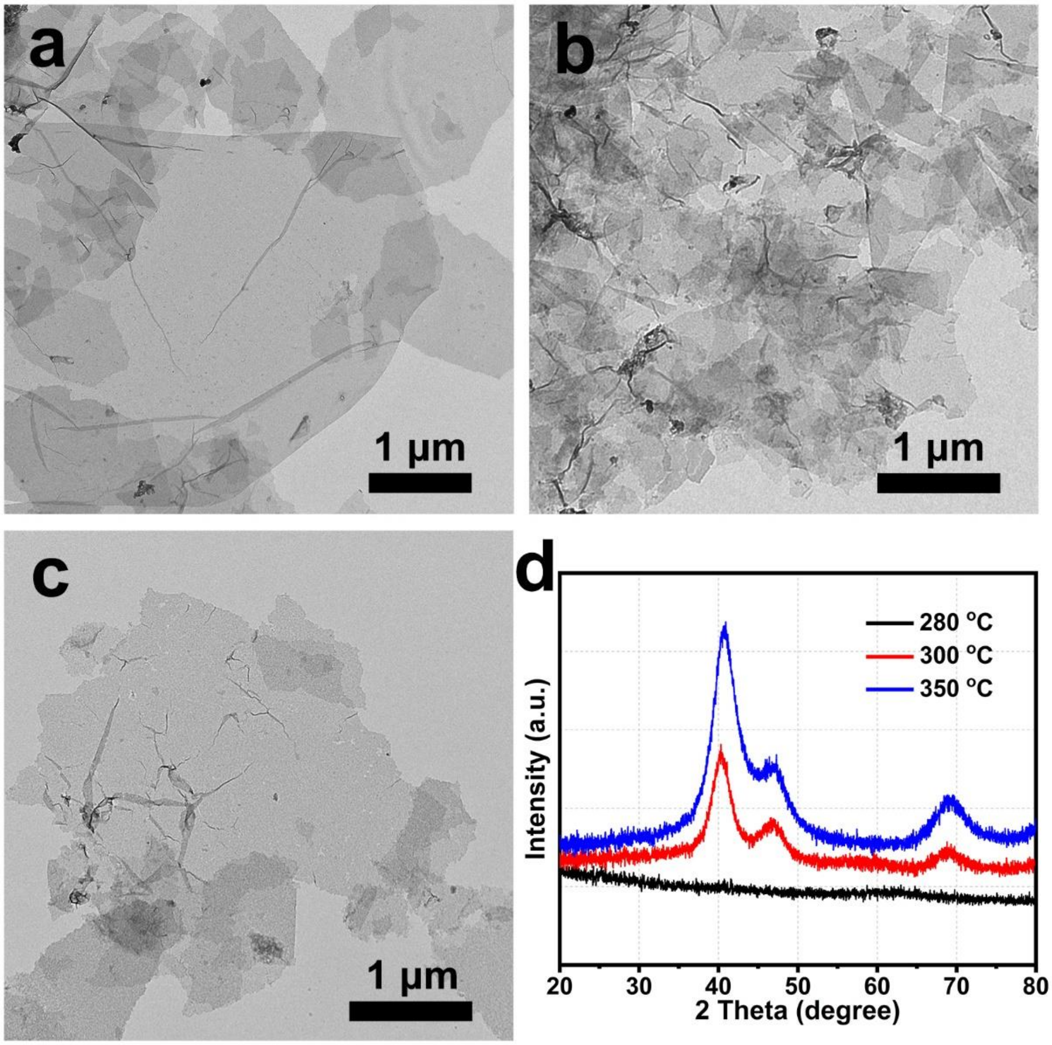

Supplementary Figure 1. Characterizations of Ir nanosheets obtained at different annealing temperature. (a, b, c) TEM images of nanosheets obtained by annealing at 280 °C, 300 °C and 350 °C, respectively. (d) XRD pattern of nanosheets obtained.

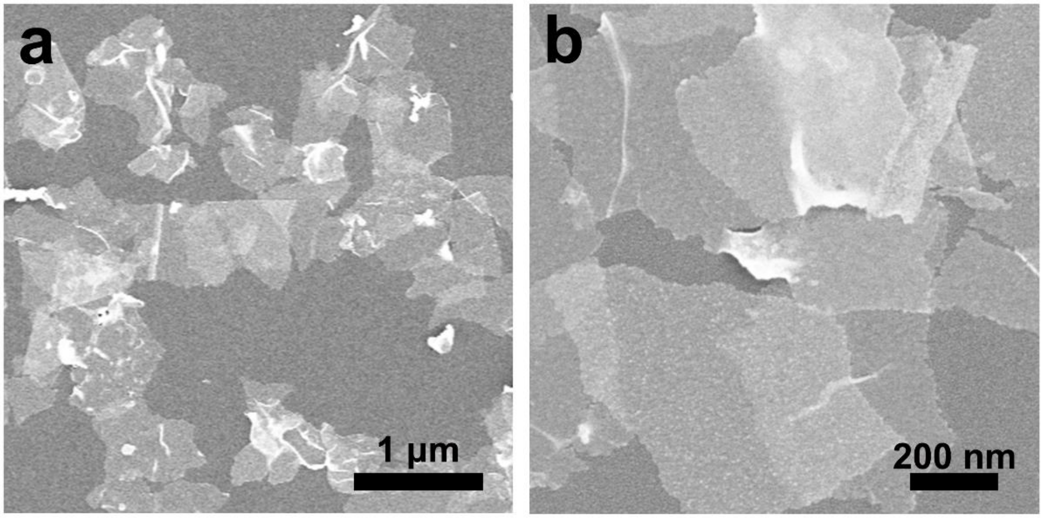

Supplementary Figure 2. SEM characterization of AC-Ir NSs. (a, b) SEM images of AC-Ir NSs with different resolution.

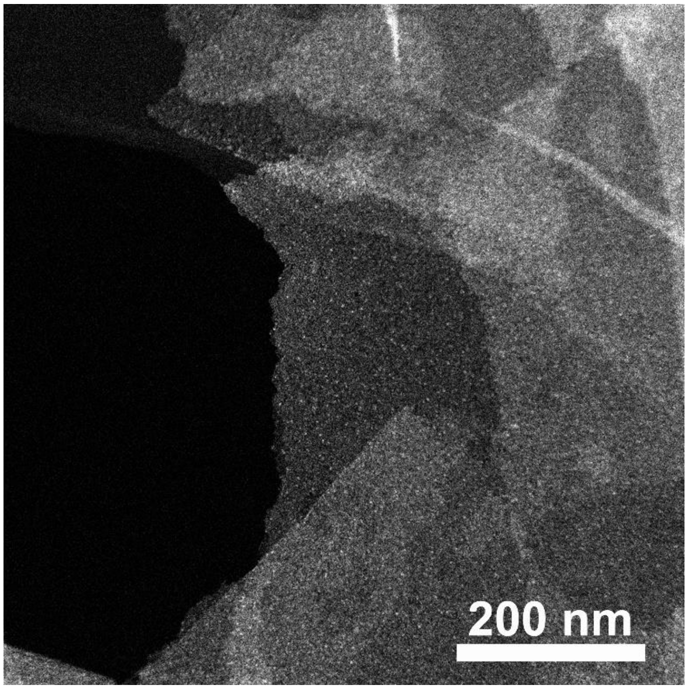

Supplementary Figure 3. HAADF-STEM characterization of AC-Ir NSs. HAADF-STEM image of AC-Ir NSs.

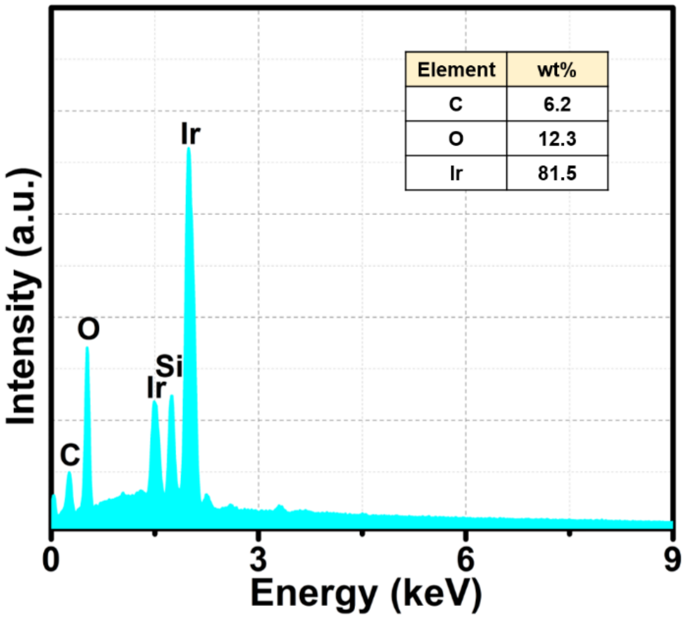

Supplementary Figure 4. The compositional analysis of AC-Ir NSs. EDS spectrum of AC-Ir NSs.

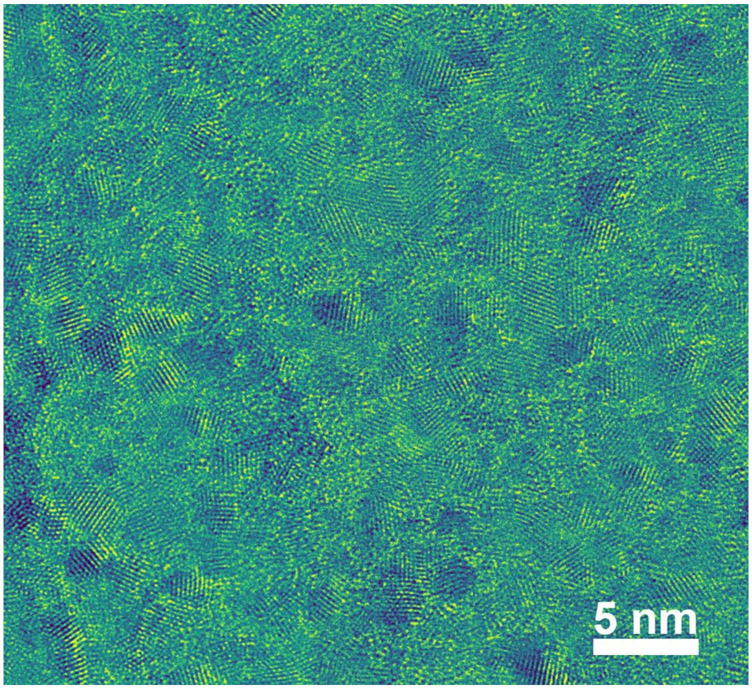

Supplementary Figure 5. HRTEM characterization of AC-Ir NSs. HRTEM image with false color of AC-Ir NSs at different area.

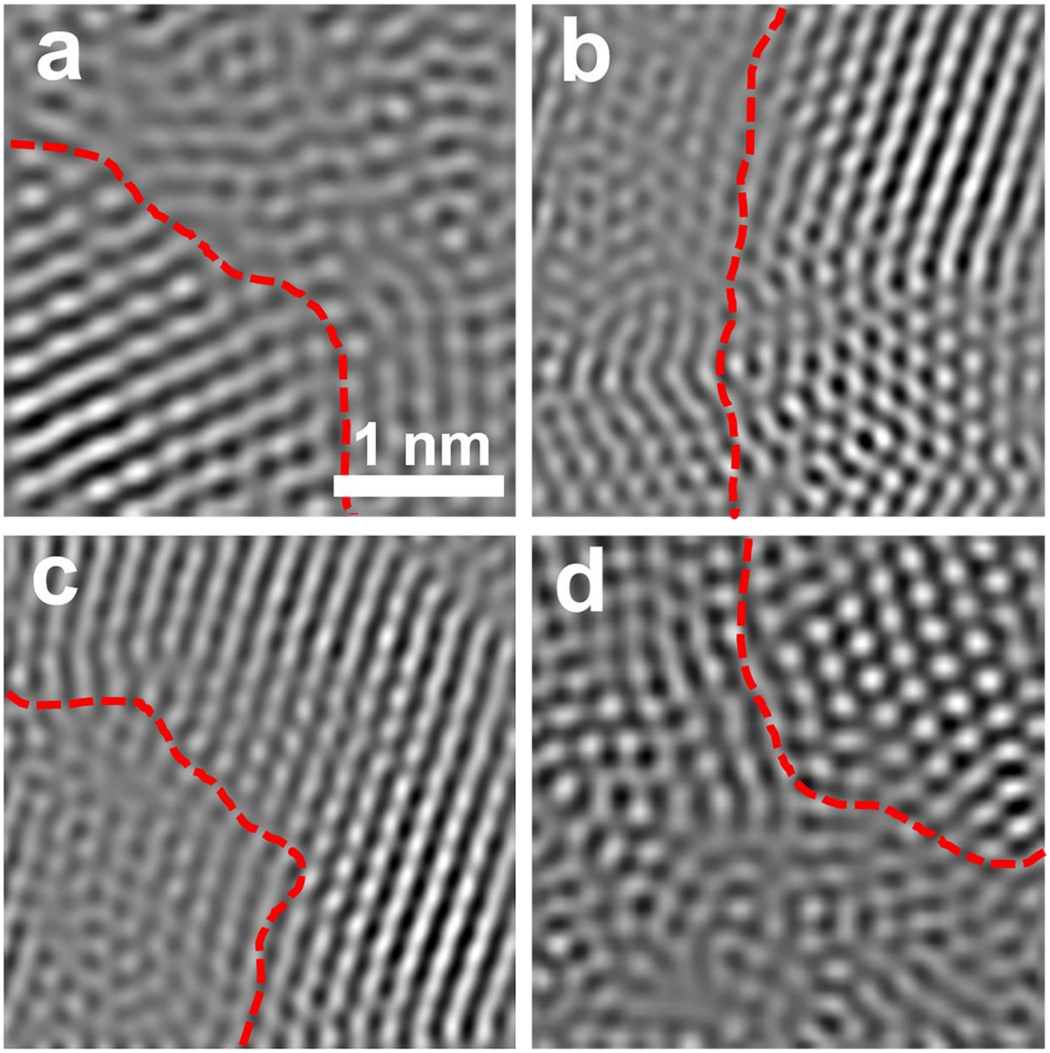

Supplementary Figure 6. HRTEM characterization of amorphous-crystalline phase boundary. (a, b, c, d) HRTEM images of amorphous-crystalline phase boundary at different areas.

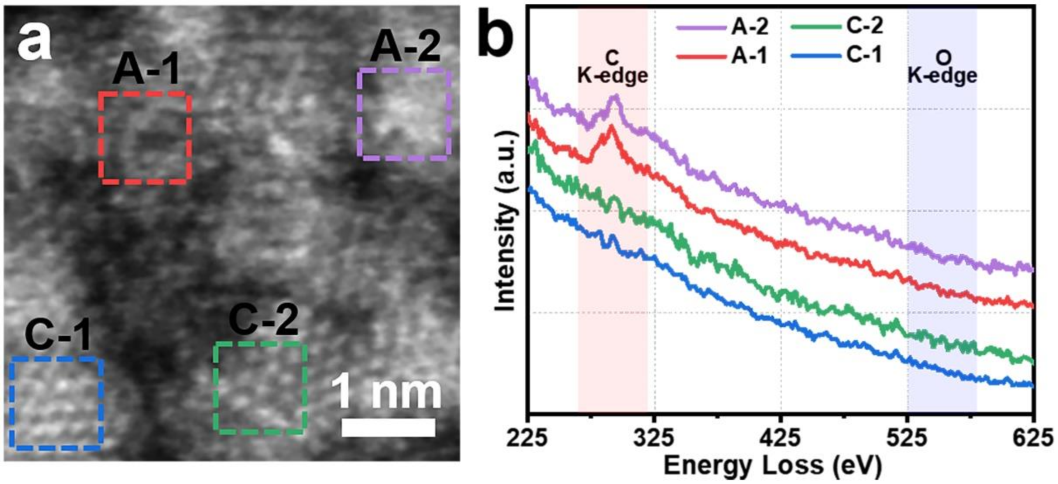

Supplementary Figure 7. High-resolution EELS analysis around amorphous-crystalline phase boundary. (a) Aberration-corrected HAADF-STEM image around amorphous-crystalline phase boundary at different area. (b) EELS spectrum of amorphous domains and crystalline domains corresponding to the selected area in (a), respectively.

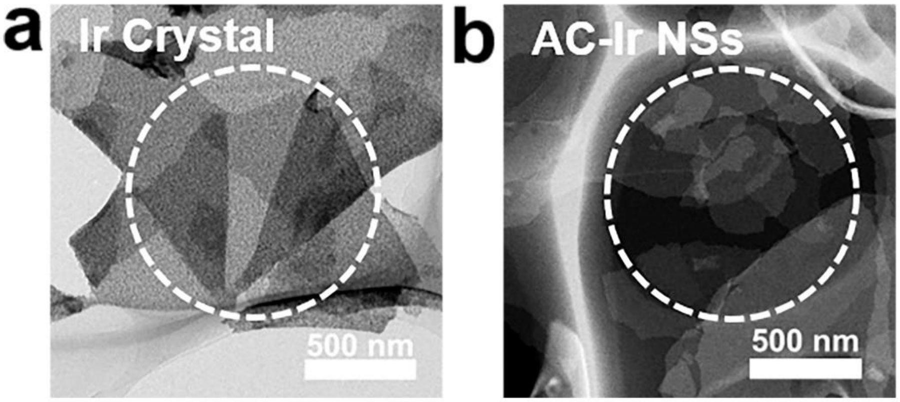

Supplementary Figure 8. Selected area of SAED for Ir crystal and AC-Ir NSs. (a, b) Selected area (white dotted circle) of SAED for Ir crystal and AC-Ir NSs, respectively.

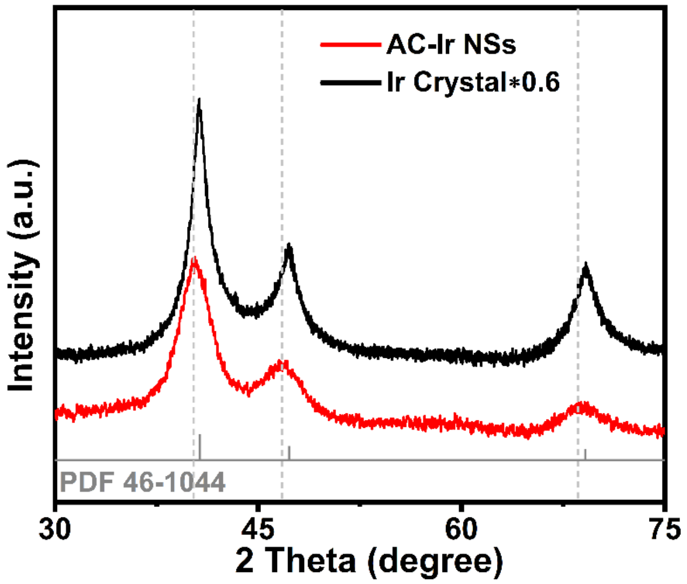

Supplementary Figure 9. XRD pattern of AC-Ir NSs. XRD pattern of AC-Ir NSs and Ir crystal, respectively.

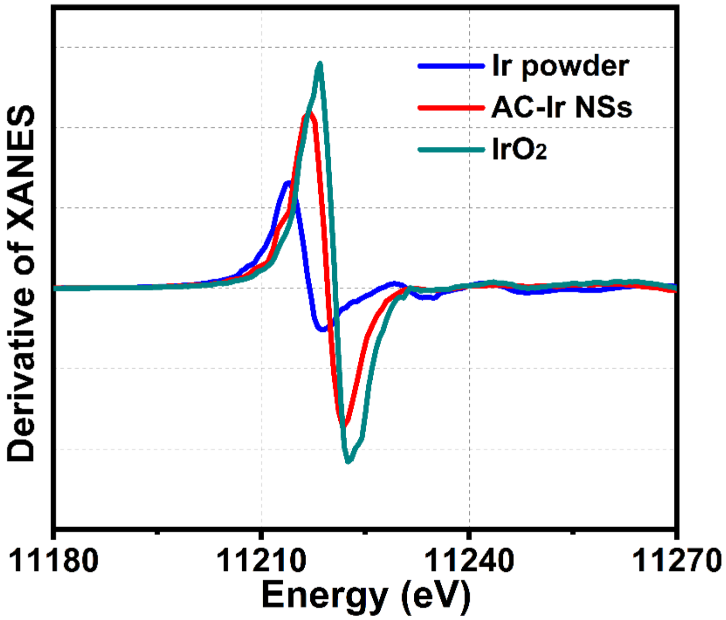

Supplementary Figure 10. The first derivative XANES spectrum of AC-Ir NSs. The first derivative XANES spectrum of Ir powder, IrO_2 and AC-Ir NSs, respectively.

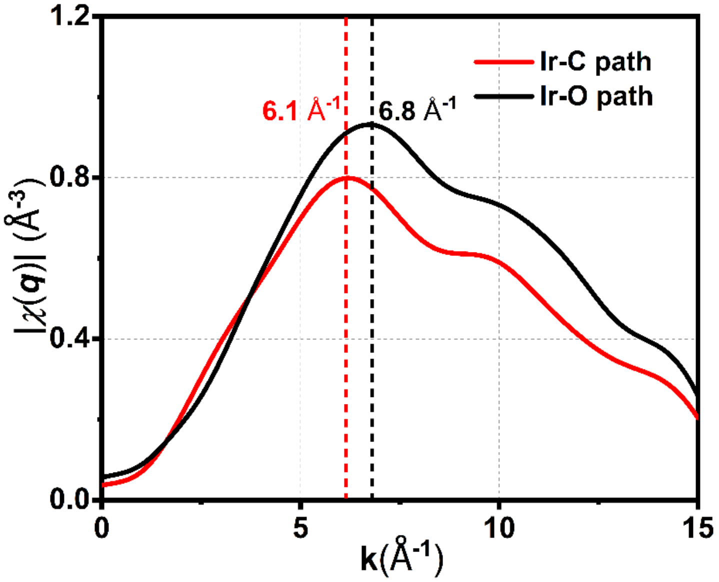

Supplementary Figure 11. The FEFF-calculated q-space magnitudes for Ir-C path and Ir-O path. Comparison of the FEFF-calculated q-space magnitudes for Ir-C path and Ir-O path, respectively.

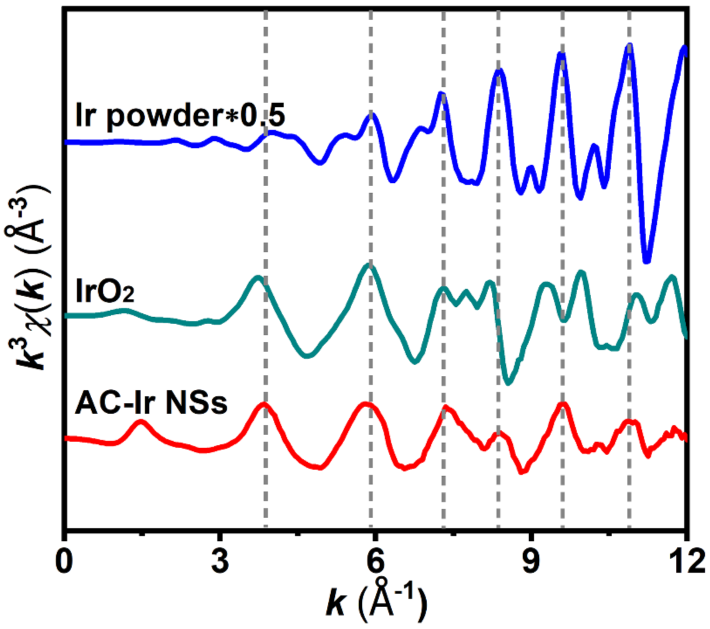

Supplementary Figure 12. The Ir L_3-edge $k^3\chi(k)$ oscillations curves of AC-Ir NSs. The Ir L_3-edge $k^3\chi(k)$ oscillations curves of Ir powder, IrO_2 and AC-Ir NSs, respectively.

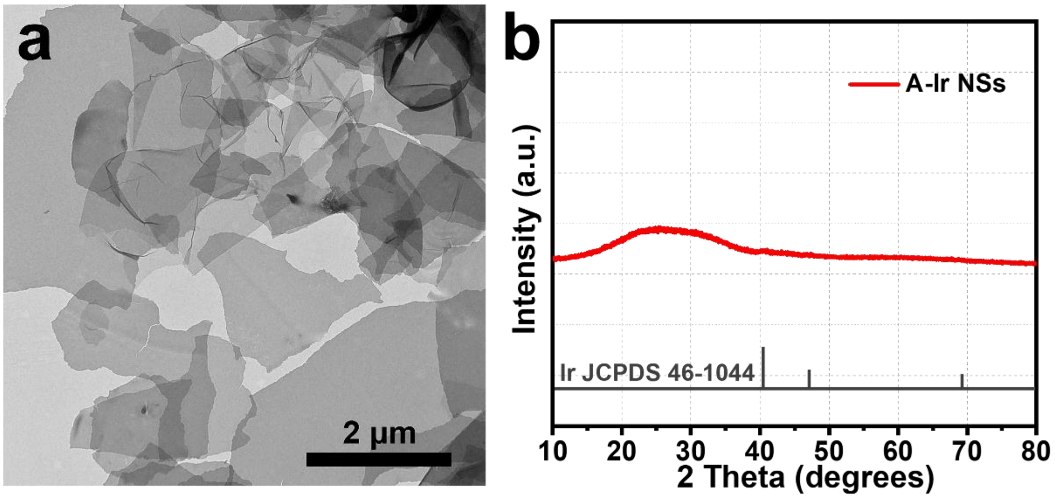

Supplementary Figure 13. Characterizations of A-Ir NSs. (a) TEM image and (b) XRD pattern of A-Ir NSs.

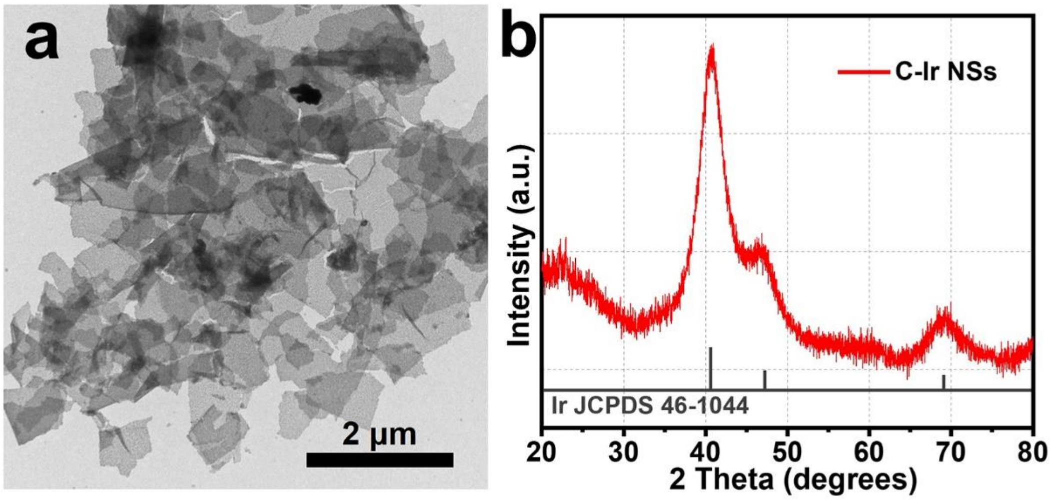

Supplementary Figure 14. Characterizations of C-Ir NSs. (a) TEM image, (b) XRD pattern of C-Ir NSs.

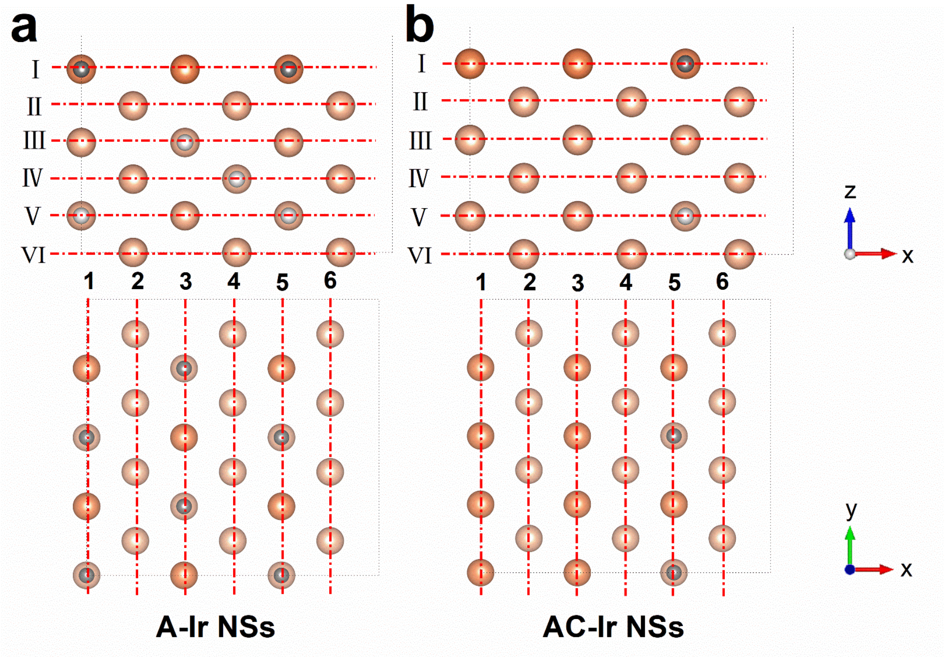

Supplementary Figure 15. The side and top views of A-Ir NSs and AC-Ir NSs. (a, b) The side and top views of A-Ir NSs and AC-Ir NSs, respectively. Note: Orange, grey, light orange and light grey spheres represent surface iridium, surface carbon, subsurface iridium and subsurface carbon atoms in the slab models, respectively.

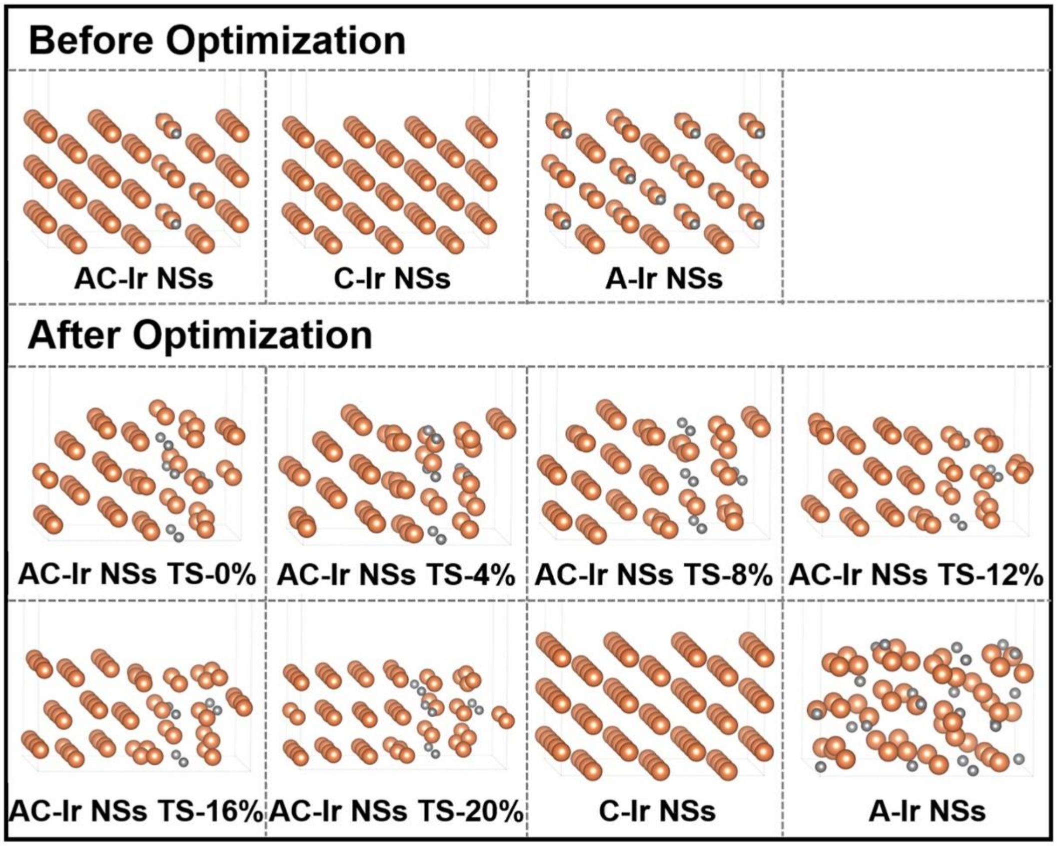

Supplementary Figure 16. The slab models of C-Ir NSs, A-Ir NSs and AC-Ir NSs. The slab models of C-Ir NSs, A-Ir NSs and AC-Ir NSs before and after optimization. Note: The size and shape of slab model is a periodic six-layer slab repeated in 3×2 surface unit cell with a vacuum region of 12 Å between the slabs along the z axis. Orange and grey spheres in the slab models represent iridium and carbon atoms.

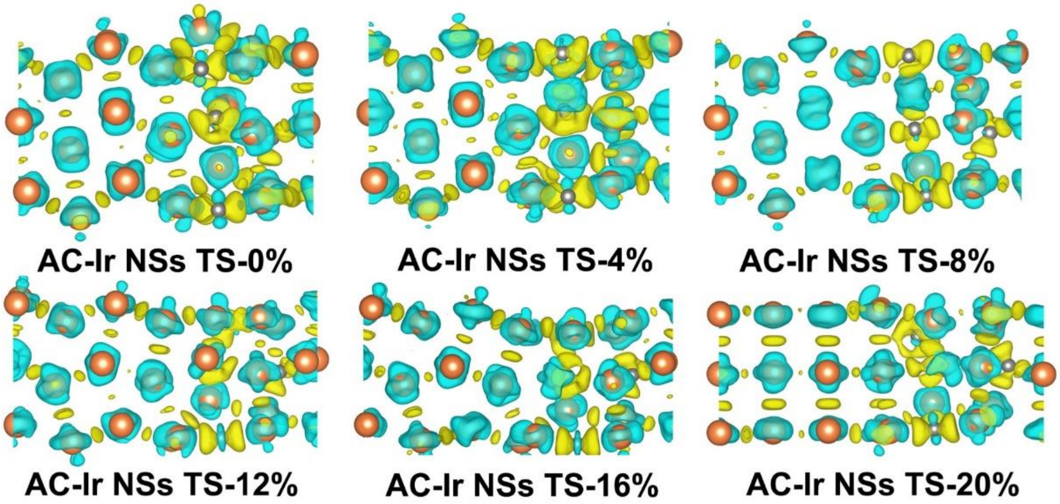

Supplementary Figure 17. The deformation charge density analysis of AC-Ir NSs. The deformation charge density analysis of AC-Ir NSs with different degrees of lattice expansion (isosurface is 0.12 bohr^{-3}, cyan represent charge depletion and yellow represent charge accumulation.). Note: Orange and grey spheres in the slab models represent Ir and C atoms.

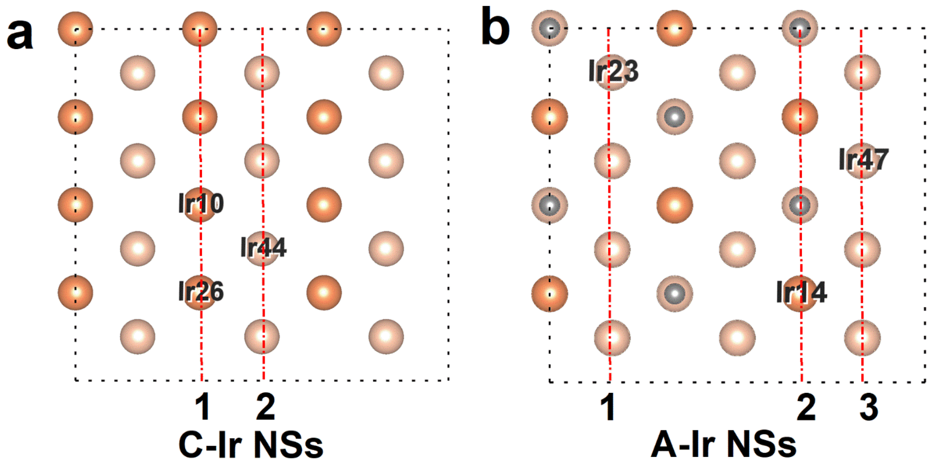

Supplementary Figure 18. The schematic sites of the H* adsorption on C-Ir NSs and A-Ir NSs. (a, b) The schematic sites of the H* adsorption on C-Ir NSs and A-Ir NSs from the top view, respectively. Note: Orange, grey, light orange and light grey spheres represent surface iridium, surface carbon, subsurface iridium and subsurface carbon atoms in the slab models, respectively.

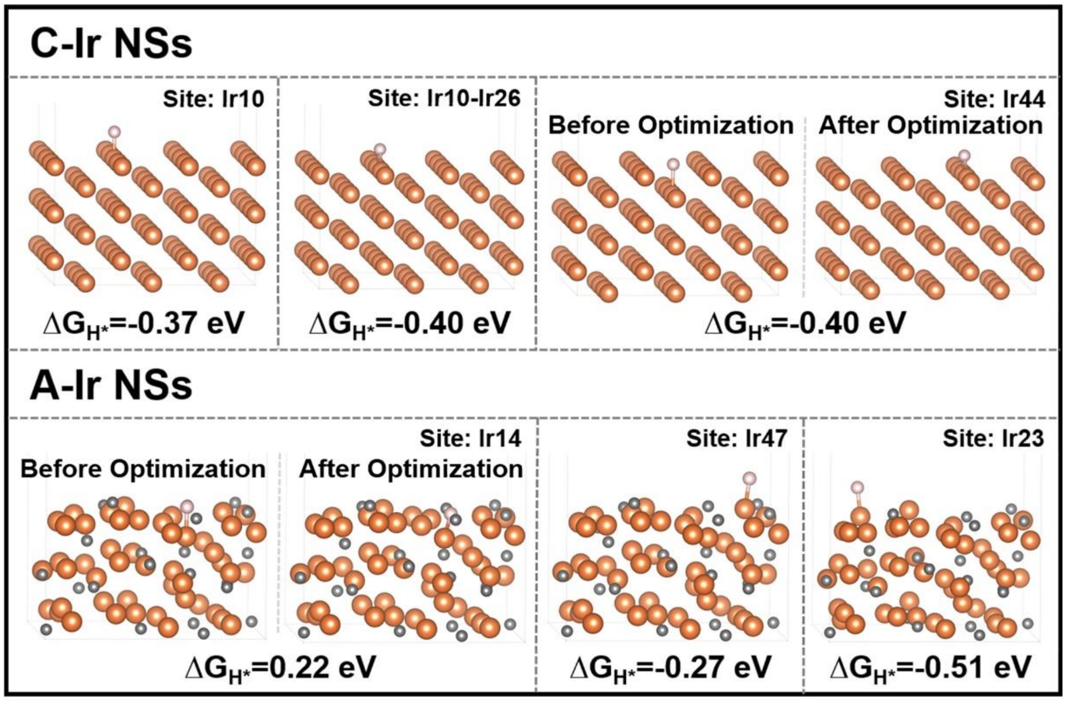

Supplementary Figure 19. The structures of the H^* adsorption on C-Ir NSs and A-Ir NSs. The structures of the H^* adsorption on C-Ir NSs and A-Ir NSs. Note: Two structures, before and after optimization, are shown in those cases when the adsorption position of H^* changed during the optimization. Orange, grey and light red spheres in the slab models represent iridium, carbon and hydrogen atoms, respectively. The number is denoted as the location marked in Supplementary Fig. 18.

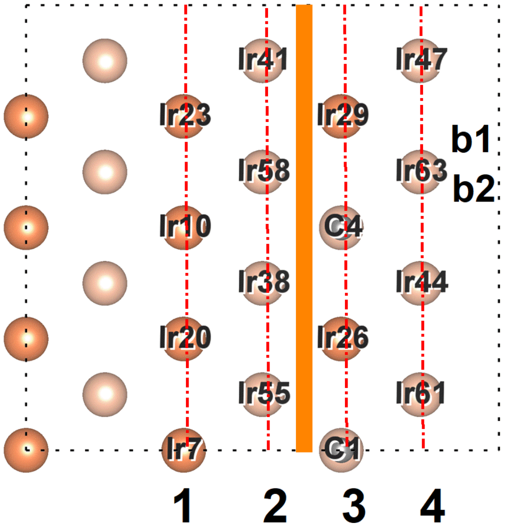

Supplementary Figure 20. The schematic H* adsorption sites on AC-Ir NSs. The schematic sites of the H* adsorption on AC-Ir NSs from the top view. Note: the interface between column 2 and 3 is considered as the amorphous-crystalline boundary. Orange, grey, light orange and light grey spheres represent surface iridium, surface carbon, subsurface iridium and subsurface carbon atoms in the slab models, respectively.

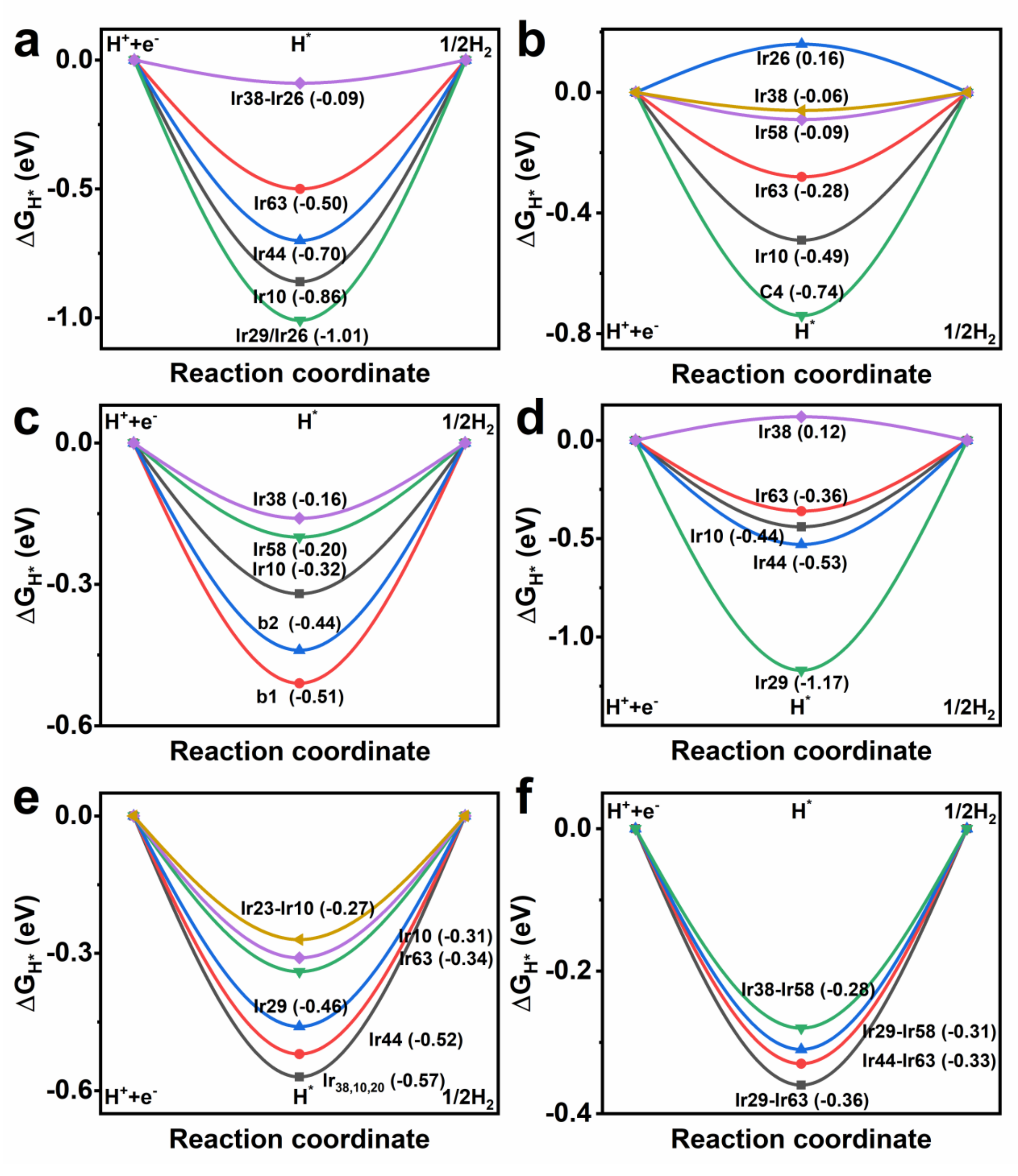

Supplementary Figure 21. The calculated ΔG_{H^*} on different Ir active sites of AC-Ir NSs. The calculated ΔG_{H^*} on different Ir active sites of AC-Ir NSs with different degrees of lattice expansion: (a) 0% tensile strain, (b) 4% tensile strain, (c) 8% tensile strain, (d) 12% tensile strain, (e) 16% tensile strain and (f) 20% tensile strain, respectively. Note: the number is denoted as the location marked in Supplementary Fig. 20.

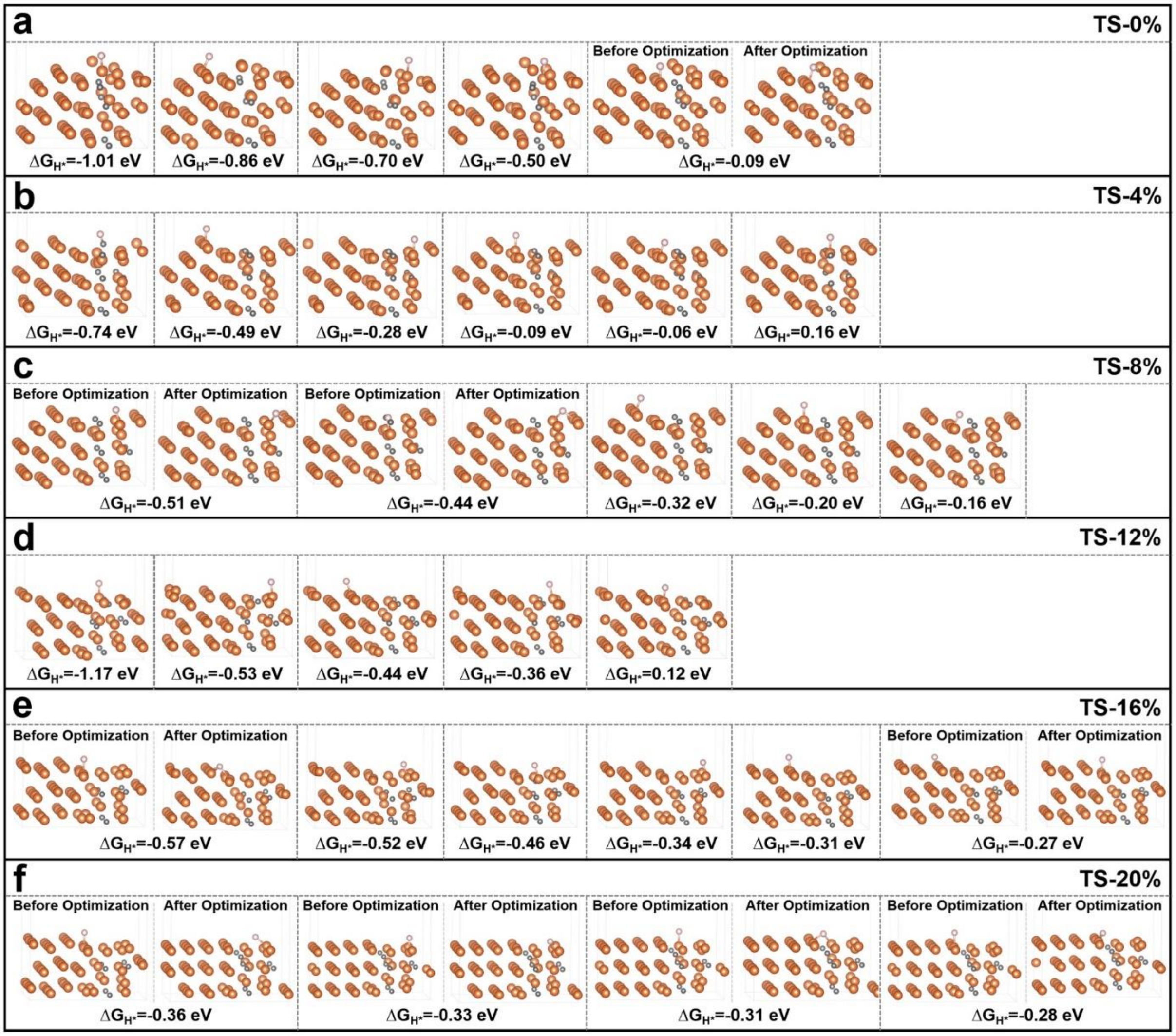

Supplementary Figure 22. The structures of the H^* adsorption on AC-Ir NSs. The structures of the H^* adsorption on AC-Ir NSs with different degrees of lattice expansion: (a) 0% tensile strain, (b) 4% tensile strain, (c) 8% tensile strain, (d) 12% tensile strain, (e) 16% tensile strain and (f) 20% tensile strain, respectively, which are corresponding to the energy diagrams of Supplementary Fig. 21. Note: Two structures, before and after optimization, are shown in those cases when the adsorption position of H^* changed during the optimization. Orange, grey and light red spheres in the slab models represent iridium, carbon and hydrogen atoms, respectively.

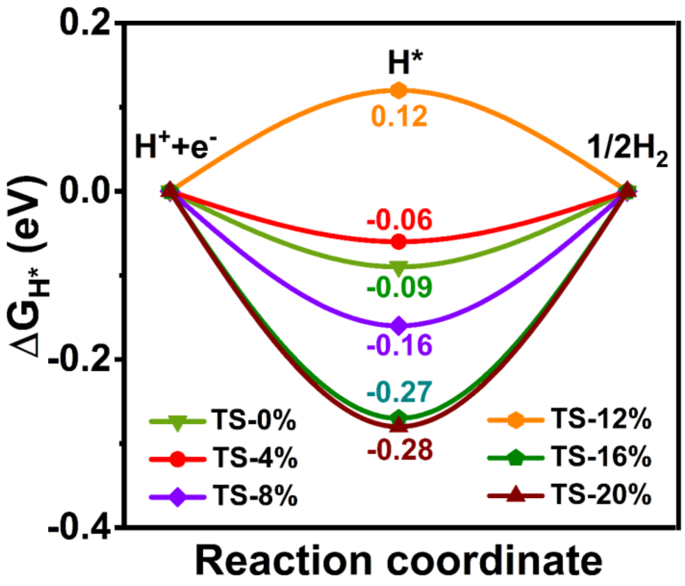

Supplementary Figure 23. The optimal ΔG_{H^*} of AC-Ir NSs. The optimal ΔG_{H^*} comparison between interfacial models with different degrees of lattice expansion. Note: In the DFT calculation of HER catalysis, since the ΔG_{H^*} close to neutral is extensively considered as the favourable H^* adsorption, the minimum absolute value is considered as the optimal ΔG_{H^*} for each degree of strain.

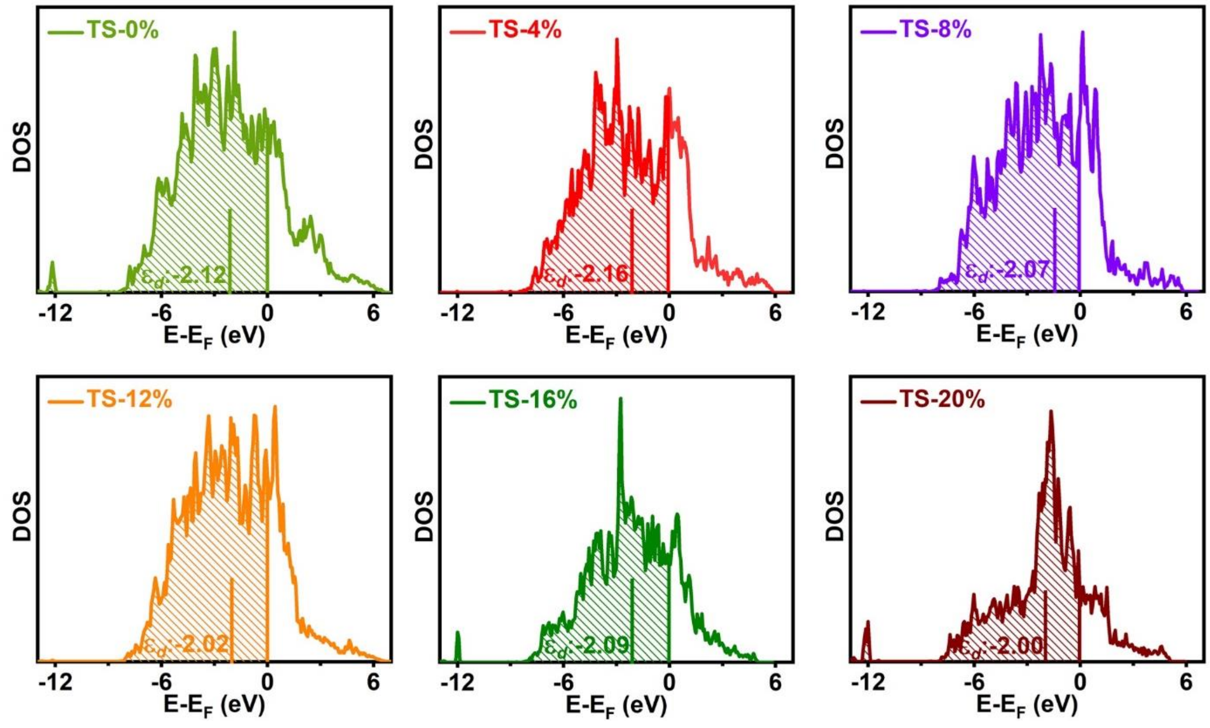

Supplementary Figure 24. The $d_{xz+yz+z^2}$ band centers of the active Ir sites for AC-Ir NSs. The $d_{xz+yz+z^2}$ band centers of the active Ir sites for AC-Ir NSs with different degrees of lattice expansion. Note: ε_d refers to $E_{dxz+yz+z^2}$. These results imply the strain tensile can affect the position of the $d_{xz+yz+z^2}$ band center of AC-Ir NSs.

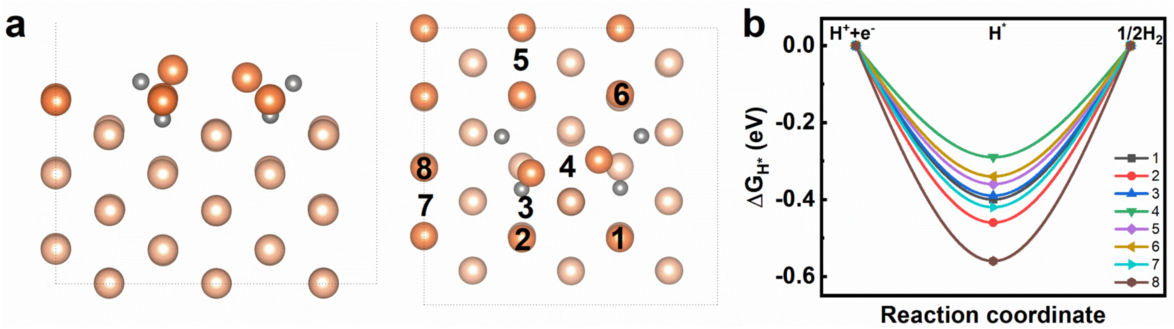

Supplementary Figure 25. The DFT calculations of the C-doped crystalline Ir nanosheets. (a) The side and top views of the optimized C-doped crystalline Ir surface. (b) The calculated ΔG_{H*} of C-doped crystalline Ir NSs. Note: the number is denoted as the location marked in (a). Orange, grey, light orange and light grey spheres represent surface iridium, surface carbon, subsurface iridium and subsurface carbon atoms in the slab models, respectively.

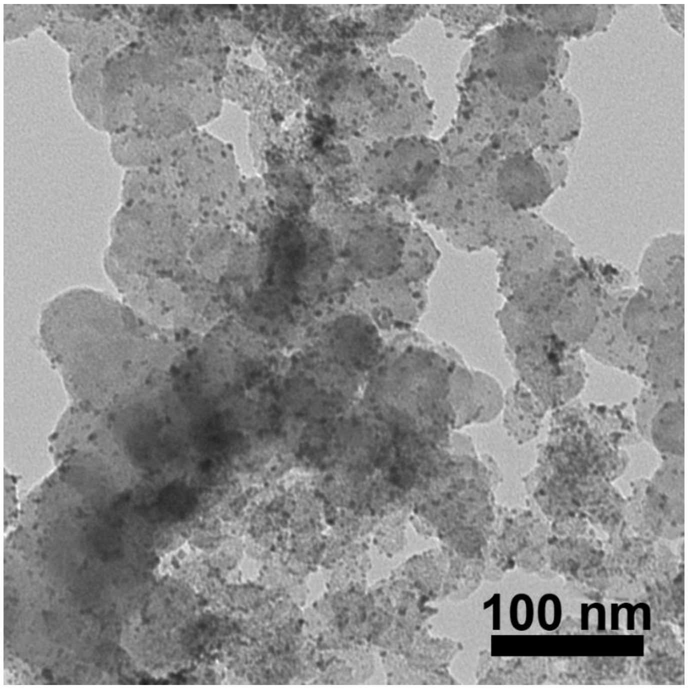

Supplementary Figure 26. Characterization of Commercial Pt/C catalysts. TEM image of commercial Pt/C catalysts.

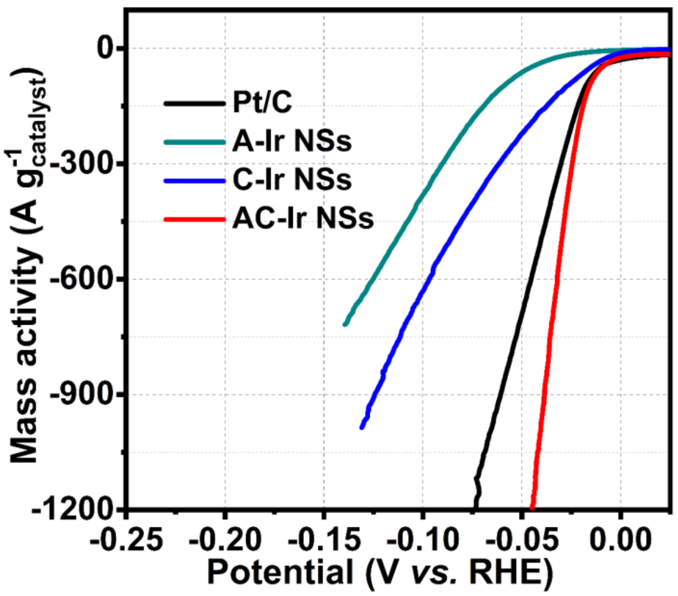

Supplementary Figure 27. Mass activity of catalysts. Mass activity of AC-Ir NSs, A-Ir NSs, C-Ir NSs and commercial Pt/C catalysts, respectively.

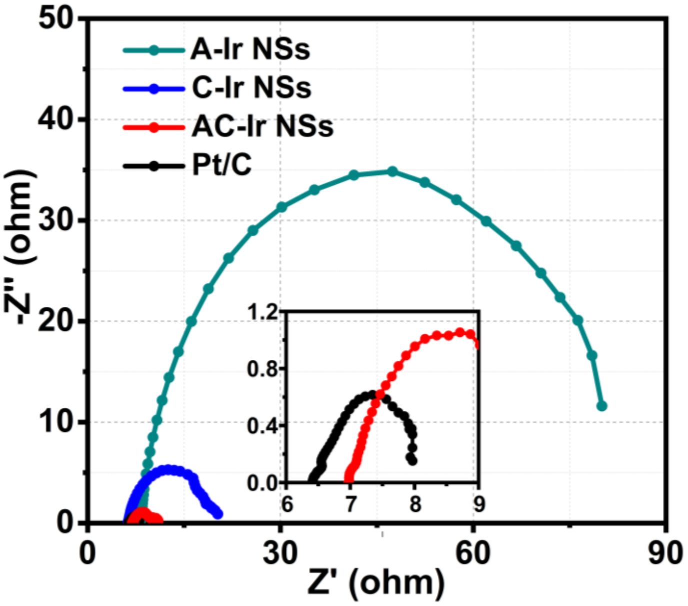

Supplementary Figure 28. Nyquist plots of catalysts. Nyquist plots of AC-Ir NSs, A-Ir NSs, C-Ir NSs and commercial Pt/C catalysts at an overpotential of 20 mV (*vs.* RHE), respectively.

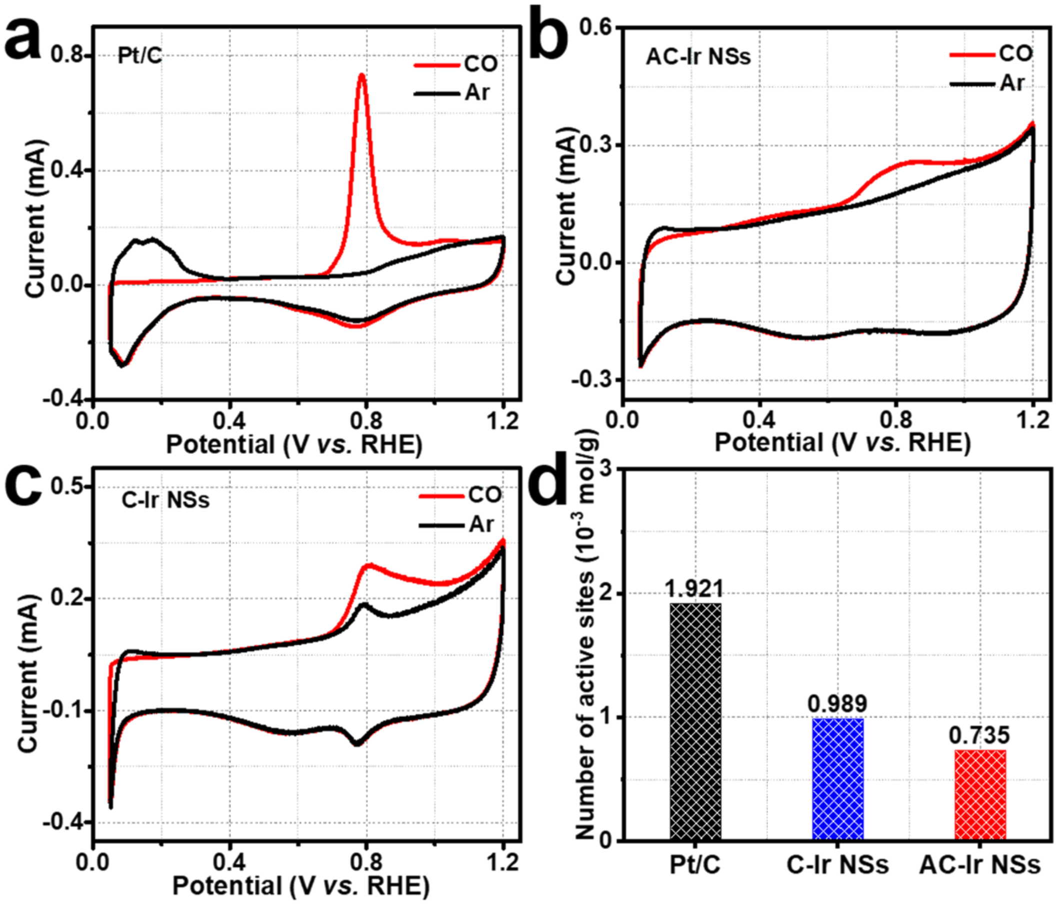

Supplementary Figure 29. Active sites calculation of catalysts. (a, b, c) CO stripping voltammetry of commercial Pt/C catalysts, AC-Ir NSs and C-Ir NSs at scan rat of 10 mV s^{-1}. Stripping of a monolayer of CO in first cycle (red), subsequently second cycle after the stripping of CO (black). (d) Estimation of the active sites of commercial Pt/C catalysts, AC-Ir NSs and C-Ir NSs, respectively. The value obtained by CO stripping may qualitatively reflect the trend of active site number of tested catalysts to some extent.

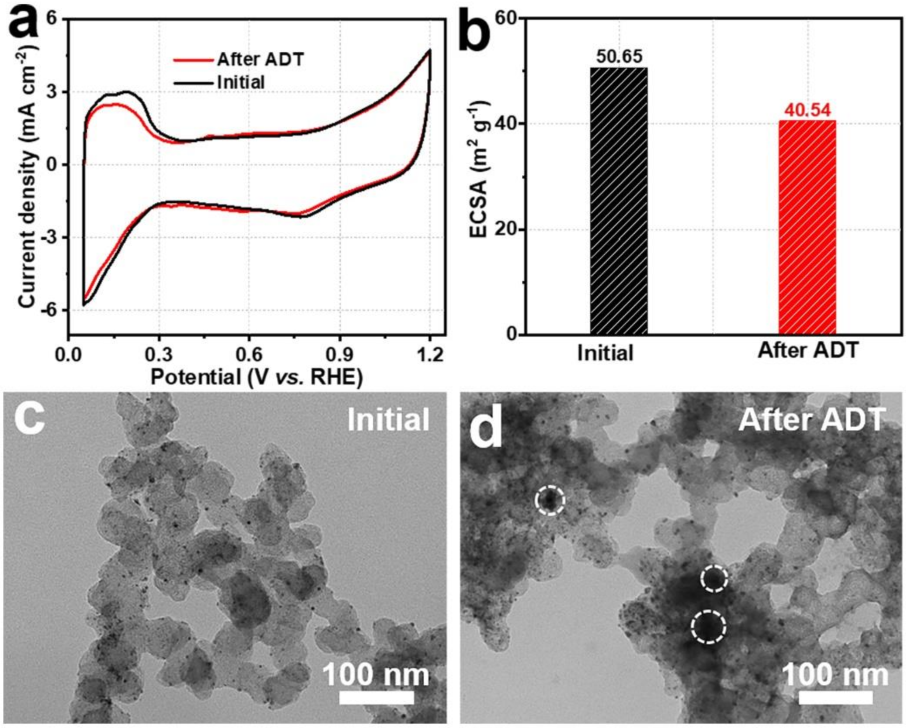

Supplementary Figure 30. Characterizations of commercial Pt/C catalysts before and after ADT. (a) Cyclic voltammetry curves, (b) The electrochemical active surface area (ECSA) and (c, d) TEM images of commercial Pt/C catalysts before and after ADT. The dotted circles in d correspond to the aggregation of Pt nanoparticles. These results reveal that the well-dispersed Pt nanoparticles in the commercial Pt/C catalysts agglomerated into irregular larger nanoparticles after ADT, suggesting the instability of commercial Pt/C catalysts during ADT.

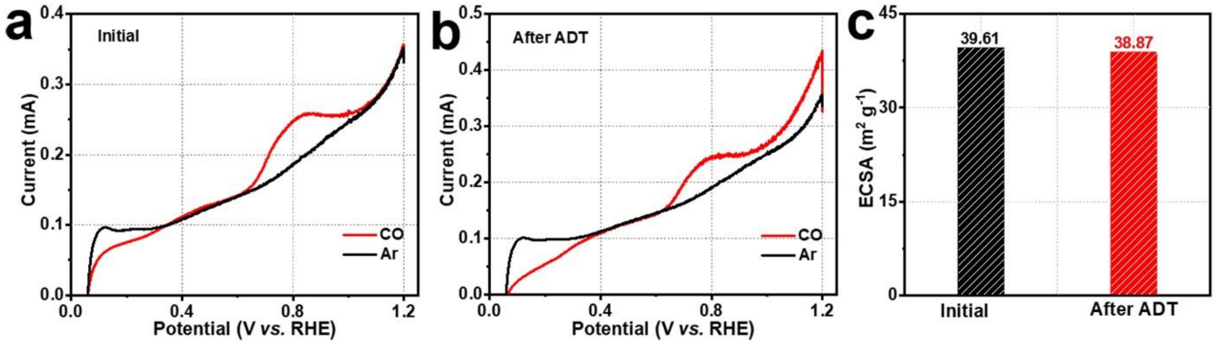

Supplementary Figure 31. The electrochemical active surface area of AC-Ir NSs before and after ADT. (a, b) CO stripping voltammetry of AC-Ir NSs before and after ADT. Stripping of a monolayer of CO in first cycle (red), subsequently second cycle after the stripping of CO (black). (c) Estimation of electrochemical active surface area (ECSA) for AC-Ir NSs before and after ADT. The ECSA of AC-Ir NSs shows ignorable change after ADT, revealing AC-Ir NSs are electrochemically robust.

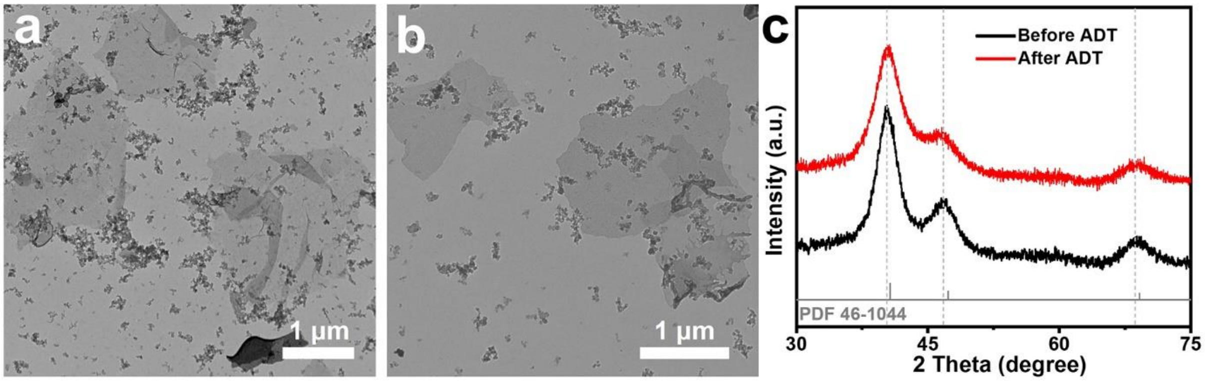

Supplementary Figure 32. Characterizations of AC-Ir NSs before and after ADT. (a, b) TEM images before and after ADT, respectively. (c) XRD pattern before and after ADT, respectively.

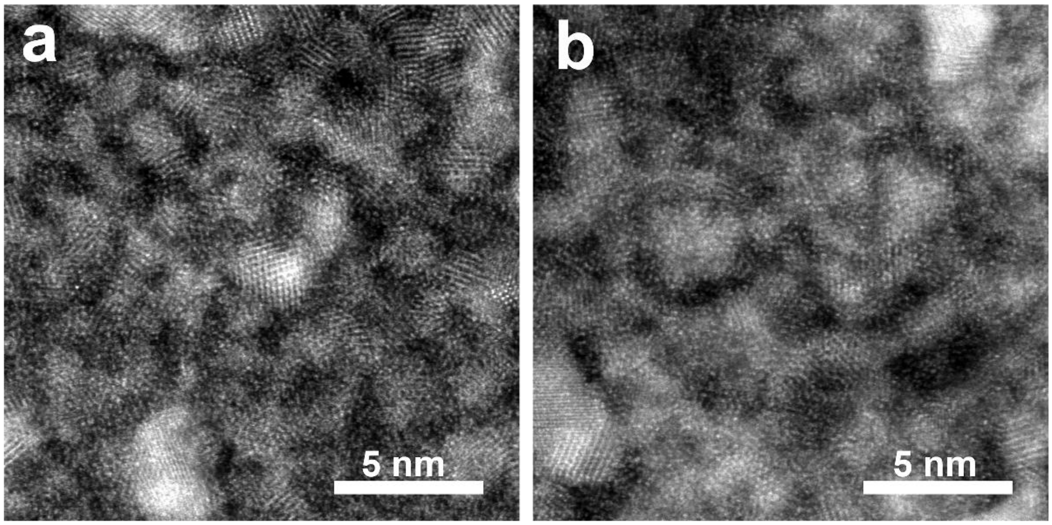

Supplementary Figure 33. Aberration-corrected HAADF-STEM characterizations of AC-Ir NSs after ADT. (a, b) Aberration-corrected HAADF-STEM images of AC-Ir NSs at different areas after ADT.

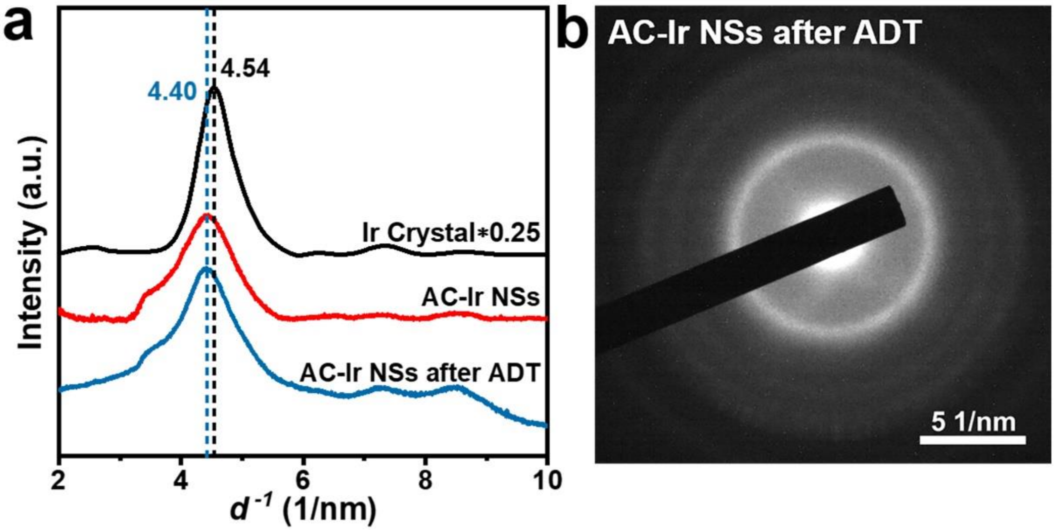

Supplementary Figure 34. Electron diffraction profile of AC-Ir NSs after ADT. (a) Electron diffraction profile and (b) selected area electron diffraction pattern of AC-Ir NSs after ADT. The position of main peak of AC-Ir NSs after ADT remains unchanged, suggesting the tensile strain of AC-Ir NSs is electrochemically stable during HER catalysis.

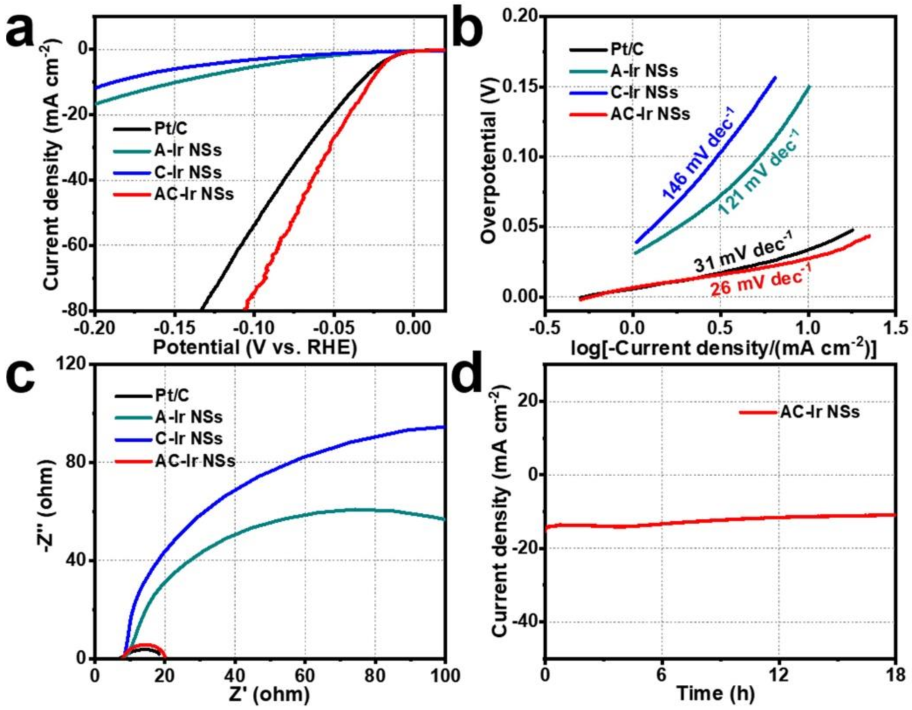

Supplementary Figure 35. Electrochemical HER performance of AC-Ir NSs in alkaline medium. (a) Polarization curves and (b) Tafel plots of AC-Ir NSs, A-Ir NSs, C-Ir NSs and commercial Pt/C catalysts, respectively. (c) Nyquist plots of catalysts at an overpotential of 20 mV (*vs.* RHE). (d) Chronoamperometry measurement at an overpotential of 28 mV (*vs.* RHE) for AC-Ir NSs. Note: All the measurements were conducted in N_2-saturated 1.0 M KOH solution.

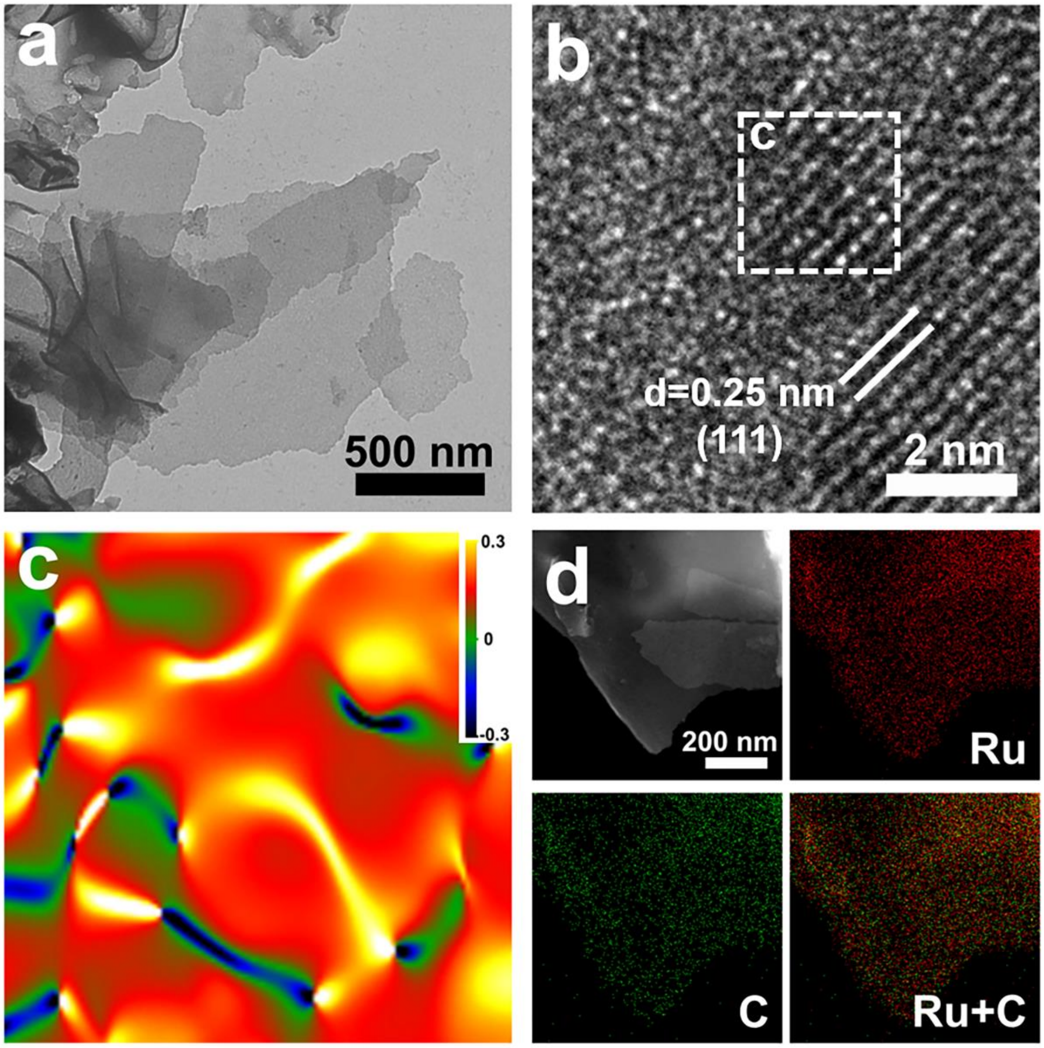

Supplementary Figure 36. Characterizations of AC-Ru NSs. (a) TEM image, (b) HRTEM image of AC-Ru NSs. (c) e_{yy} strain component of crystalline domain acquired by GPA method. (d) EDS elemental mapping images. Note: the lattice strain e_{yy} is associated with the expansion/contraction of the lattice vector along the in-plane y direction.

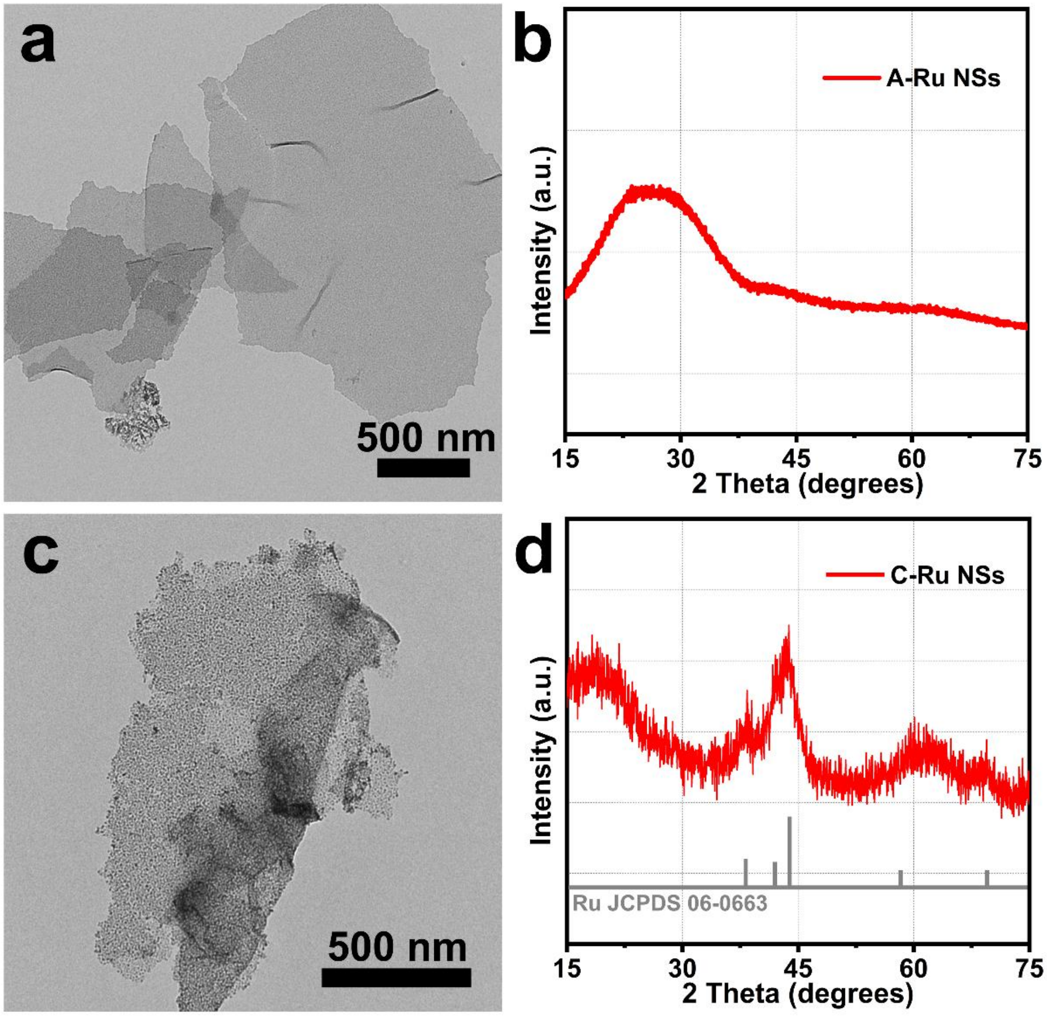

Supplementary Figure 37. Characterizations of A-Ru NSs and C-Ru NSs. (a, c) TEM images and (b, d) XRD patterns of A-Ru NSs and C-Ru NSs, respectively.

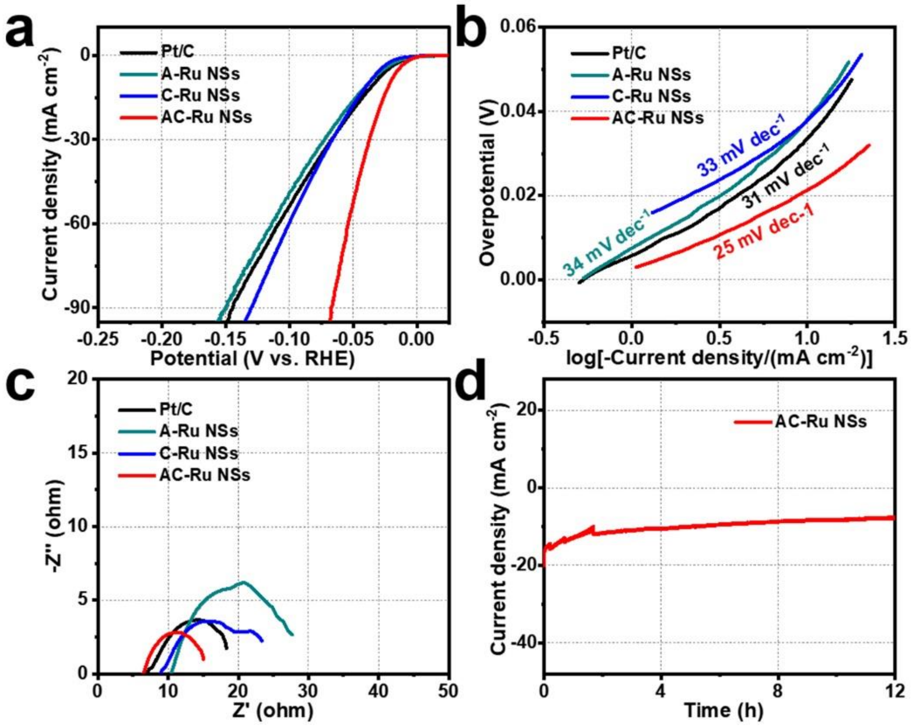

Supplementary Figure 38. Electrochemical HER performance of AC-Ru NSs. (a) Polarization curves and (b) Tafel plots of AC-Ru NSs, A-Ru NSs, C-Ru NSs and commercial Pt/C catalysts, respectively. (c) Nyquist plots of catalysts at an overpotential of 20 mV (*vs.* RHE). (d) Chronoamperometry measurement at an overpotential of 22 mV (*vs.* RHE) for AC-Ru NSs. Note: All the measurements were conducted in N_2-saturated 1.0 M KOH solution.

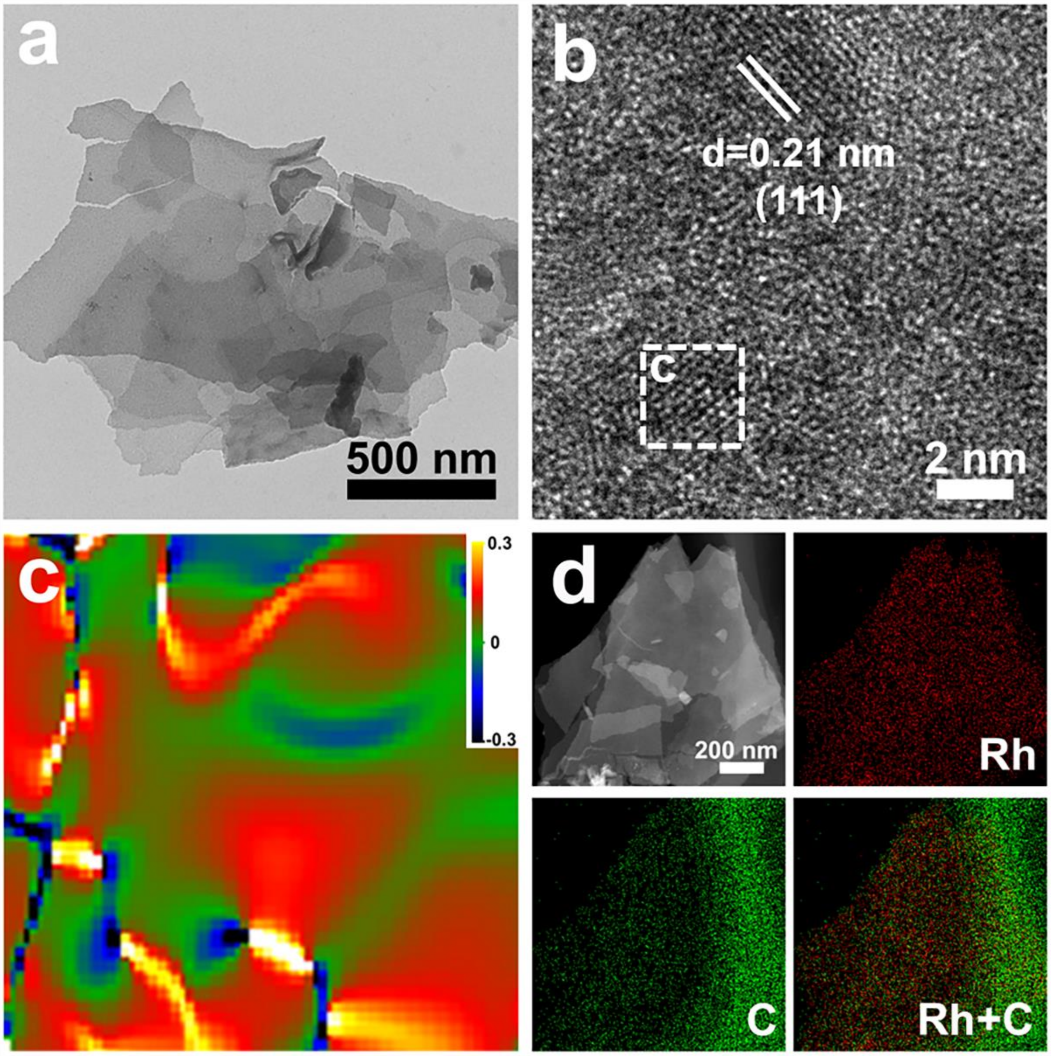

Supplementary Figure 39. Characterizations of AC-Rh NSs. (a) TEM image, (b) HRTEM image of AC-Rh NSs, (c) e_{yy} strain component of crystalline domain acquired by GPA method. (d) EDS elemental mapping images. Note: the lattice strain e_{yy} is associated with the expansion/contraction of the lattice vector along the in-plane y direction.

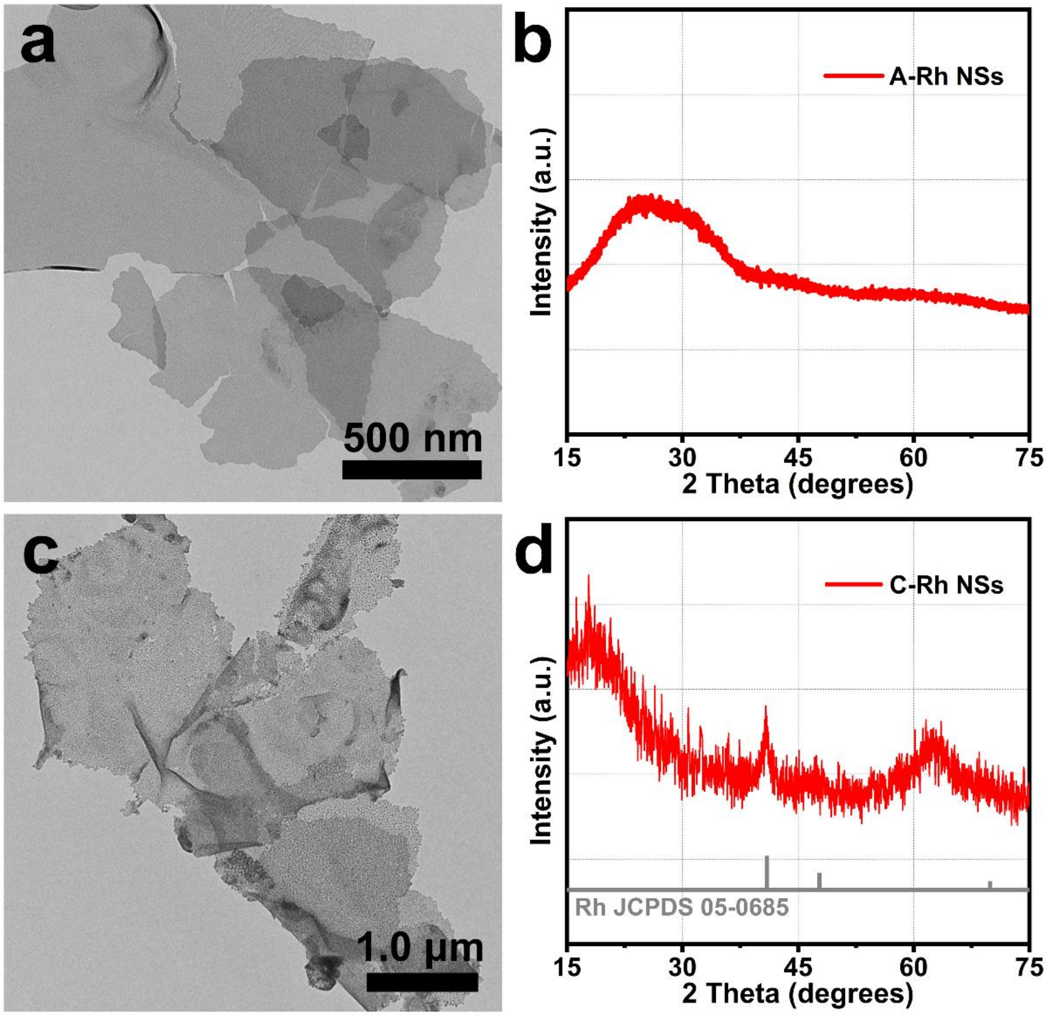

Supplementary Figure 40. Characterizations of A-Rh NSs and C-Rh NSs. (a, c) TEM images and (b, d) XRD patterns of A-Rh NSs and C-Rh NSs, respectively.

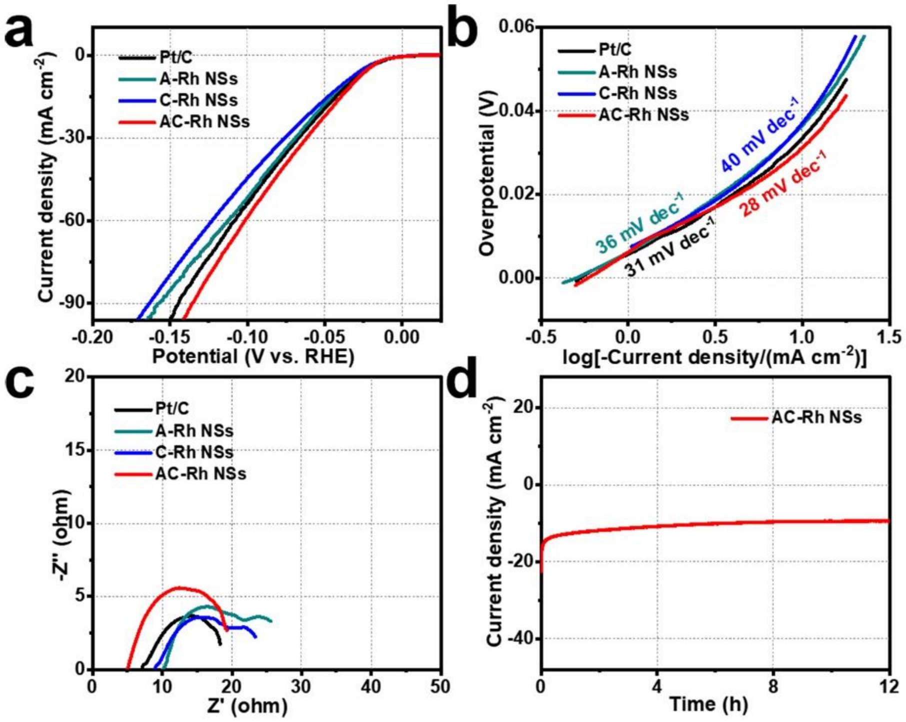

Supplementary Figure 41. Electrochemical HER performance of AC-Rh NSs. (a) Polarization curves and (b) Tafel plots of AC-Rh NSs, A-Rh NSs, C-Rh NSs and commercial Pt/C catalysts, respectively. (c) Nyquist plots of catalysts at an overpotential of 20 mV (*vs.* RHE). (d) Chronoamperometry measurement at an overpotential of 32 mV (*vs.* RHE) for AC-Rh NSs. Note: All the measurements were conducted in N_2-saturated 1.0 M KOH solution.

Supplementary Table 1. EXAFS fitting parameters at the Ir L_3-edge for Ir powder, IrO_2 and AC-Ir NS (S_0^2=0.807).

**Sample**	**Shell**	N^a	R(Å)^b	σ^2(Å^2)^c	ΔE_0(eV)^d	***R* factor**
Ir powder	Ir-Ir	12.0	2.71	0.0031	8.3	0.0012
IrO_2	Ir-O	6.0	1.98	0.0030	12.0	0.0034
	Ir-Ir	1.9	3.11	0.0028		
	Ir-O	3.8	3.43	0.0019		
	Ir-Ir	7.7	3.55	0.0047		
AC-Ir NSs	Ir-C	4.5	2.00	0.0050	9.3	0.0065
	Ir-Ir	7.2	2.90	0.0076		

Note: ^aN: coordination numbers; ^bR: bond distance; $^c\sigma^2$: Debye-Waller factors; $^d\Delta E_0$: the inner potential correction.

Supplementary Table 2. Comparison about the Bader charge analysis of AC-Ir NSs with different degrees of lattice strain.

Element	AC-Ir NSs TS-0%	AC-Ir NSs TS-4%	AC-Ir NSs TS-8%	AC-Ir NSs TS-12%	AC-Ir NSs TS-16%	AC-Ir NSs TS-20%
Ir1	8.99306	9.05594	9.06568	9.07405	9.07692	9.08944
Ir2	8.99925	9.05663	9.06082	9.11244	9.13089	9.158
Ir3	8.79488	8.95801	8.87071	8.83168	8.82962	8.79968
Ir4	8.99263	9.05522	9.06603	9.07443	9.07627	9.08848
Ir5	8.99869	9.05674	9.0608	9.11206	9.13049	9.15872
Ir6	8.79441	8.95668	8.87007	8.83091	8.82635	8.79834
Ir7	9.0379	9.07332	9.06408	9.01772	9.01321	9.07714
Ir8	9.00502	9.00611	9.0254	9.0288	9.06363	9.13283
Ir9	8.93317	8.91082	8.92061	8.92513	8.91958	8.83992
Ir10	9.03841	9.07344	9.06297	9.01795	9.01346	9.07849
Ir11	9.00455	9.00608	9.02467	9.02815	9.06394	9.13218
Ir12	8.9334	8.91142	8.92038	8.92486	8.9199	8.84015
Ir13	8.86755	8.87526	8.8201	8.75404	8.78889	8.70984
Ir14	9.05556	9.08939	9.07131	9.08041	9.06523	8.96949
Ir15	9.07056	9.06102	9.04015	9.08858	9.07963	9.1061
Ir16	8.86918	8.87506	8.8203	8.75337	8.78975	8.7086
Ir17	9.05538	9.08939	9.07107	9.07959	9.06501	8.97023
Ir18	9.07083	9.06072	9.04064	9.0887	9.07963	9.10672
Ir19	8.9894	8.97046	8.94726	8.92743	8.89626	8.83662
Ir20	9.0875	9.07765	9.07203	9.0334	9.03516	9.0224
Ir21	9.03053	9.07176	9.01675	9.03581	9.04456	9.11726
Ir22	8.98789	8.9702	8.94837	8.9279	8.89711	8.83629
Ir23	9.08629	9.07796	9.07205	9.03354	9.03545	9.02306
Ir24	9.03006	9.07154	9.01721	9.03545	9.0458	9.11753
Ir25	8.64329	8.76164	8.83657	8.71921	8.72752	8.67864
Ir26	9.04411	8.80373	8.82182	8.82133	8.79951	8.82588
Ir27	8.85999	8.85338	8.76719	8.85144	8.83145	8.82095
Ir28	8.6437	8.7615	8.83715	8.71898	8.72638	8.68103
Ir29	9.04343	8.80395	8.82107	8.8208	8.80049	8.82639
Ir30	8.86345	8.85378	8.7665	8.85125	8.83169	8.82297
Ir31	8.92963	8.92436	8.96073	8.89664	8.93092	8.84115
Ir32	9.02454	9.00093	9.00937	8.99791	9.04343	9.06494
Ir33	9.0619	9.06517	9.08004	9.05095	9.01207	8.96049
Ir34	8.92896	8.92477	8.96092	8.89655	8.92962	8.84248
Ir35	9.02596	9.00142	9.00965	8.99777	9.04354	9.06505
Ir36	9.06163	9.06518	9.07991	9.05093	9.01211	8.95968
Ir37	8.83911	8.84818	8.85057	8.96844	8.97022	8.77949
Ir38	8.84246	9.01951	9.02029	8.97187	8.90945	8.90128
Ir39	8.89431	8.8727	9.03506	9.04203	9.05204	9.10762
Ir40	8.84015	8.84673	8.85068	8.96921	8.96983	8.7802
Ir41	8.84318	9.01895	9.02091	8.97217	8.90972	8.90131
Ir42	8.89557	8.87073	9.03475	9.04139	9.05255	9.1062

Ir43	8.73873	8.75899	8.77802	8.74138	8.73818	8.83191
Ir44	8.98656	8.86951	8.80681	8.88668	8.74039	8.93778
Ir45	8.84768	8.90535	8.88454	8.89429	8.87557	8.9711
Ir46	8.74526	8.76079	8.77786	8.74264	8.73816	8.83239
Ir47	8.98521	8.86928	8.80828	8.88781	8.73715	8.9357
Ir48	8.84827	8.90677	8.88346	8.89464	8.8759	8.97335
Ir49	9.04666	8.98998	9.00295	9.01668	8.99217	9.06157
Ir50	9.05936	9.08307	9.073	9.06007	9.05627	9.10098
Ir51	8.94098	8.9004	8.91662	8.92838	8.93099	8.89865
Ir52	9.04648	8.99018	9.00299	9.01593	8.99188	9.06199
Ir53	9.05905	9.08263	9.07317	9.06091	9.05513	9.09943
Ir54	8.94045	8.89994	8.91763	8.92926	8.93124	8.89801
Ir55	8.85638	8.86695	8.87766	8.99665	9.01637	9.05913
Ir56	8.89629	8.91619	8.86687	8.89596	8.88732	8.84904
Ir57	8.98381	8.9576	8.98249	8.92946	8.92942	8.8252
Ir58	8.85701	8.8666	8.8778	8.99671	9.015	9.05795
Ir59	8.89521	8.91669	8.86518	8.89463	8.88956	8.84697
Ir60	8.98408	8.95685	8.98212	8.92997	8.92925	8.82613
Ir61	8.82547	8.81124	8.90068	8.89091	9.1413	9.10271
Ir62	8.89068	8.82189	8.8989	8.89324	8.87676	8.87973
Ir63	8.82437	8.81068	8.90131	8.89095	9.14217	9.10089
Ir64	8.88364	8.82362	8.89869	8.89264	8.87418	8.88067
C1	4.49519	4.49331	4.4537	4.46639	4.46857	4.41011
C2	4.45866	4.43557	4.34856	4.32821	4.34374	4.4042
C3	4.46218	4.3782	4.48423	4.42368	4.40756	4.41756
C4	4.49662	4.49375	4.45363	4.46702	4.46855	4.40971
C5	4.45927	4.43318	4.35063	4.32963	4.34166	4.40256
C6	4.46242	4.37856	4.48538	4.42376	4.40838	4.41776
C7	4.50644	4.45779	4.3677	4.41811	4.37841	4.41204
C8	4.50613	4.45701	4.36645	4.41815	4.37952	4.41149
Δq	1.52	0.7	0.7	0.67	0.65	1.39

Note: The Bader charge of each atom for AC-Ir NSs with different degrees of lattice strain was calculated. Here Ir1-Ir24, Ir31-Ir42, Ir49-Ir60 are belong to crystalline domain. Ir25-Ir30, Ir43-Ir48, Ir61-Ir64 and C1-C8 are belong to amorphous domain. The calculated Bader charge transfer (Δq) from crystalline domains to amorphous domains of AC-Ir NSs with tensile strain from 0% to 20% are 1.52, 0.7, 0.7, 0.67, 0.65, 1.39 eV, respectively.

Supplementary Table 3. Summary of some recently reported representative HER electrocatalysts in acidic electrolytes.

**Catalysts**	**Catalyst loading amount ($mg\ cm^{-2}$)**	**Overpotential (mV) at 10 $mA\ cm^{-2}$**	**Tafel slope ($mV\ dec^{-1}$)**	**Ref.**
**AC-Ir NSs**	81 $\mu g_{Ir}\ cm^{-2}$	**17**	**27**	**This work**
**Pt/C**	81$\mu g_{pt}\ cm^{-2}$	**20**	**29**	**This work**
Ir@CON	0.50	13.4	27	*1*
IrCo-PHNC	10.0 $\mu g_{Ir}\ cm^{-2}$	21	26.6	*2*
Ru@C_2N	0.285	22	30	*3*
Pd/Cu-Pt	41$\mu g_{(Pd+Pt)}\ cm^{-2}$	22.8	25	*4*
RhP_2@PNC	---	38	38	*5*
Rh-MoS_2	0.309	47	24	*6*
Ru-MoO_2	0.57	55	44	*7*
Pt@NHPCP	2.0 $\mu g_{pt}\ cm^{-2}$	57	27	*8*
Rh/Si NW	0.193	80	24	*9*
Pt ML Ag NF/Ni foam	---	70	53	*10*
Pt-MoS_2	75 $\mu g_{pt}\ cm^{-2}$	53	40	*11*

Supplementary Table 4. TOF values of some recently reported representative HER electrocatalysts in acidic electrolytes (0.5 M H_2SO_4).

Catalysts	Overpotential (mV)	TOF per active site (H_2 s^{-1})	Ref.
**AC-Ir NSs**	**30**	**3.61**	**This work**
	**25**	**2.21**	
**C-Ir NSs**	**30**	**0.60**	**This work**
	**25**	**0.47**	
**Pt/C**	**30**	**0.81**	**This work**
	**25**	**0.55**	
Ir@CON	25	0.66	*1*
Ru@C_2N	25	0.67	*3*
Ni_5P_4	100	3.5	*12*
Ni_2P	200	0.064	
MoS_2/Au	100	1	*13*
CoNx/C	100	0.39	*14*
CoP NPs	100	0.046	*15*
$[Mo_3S_{13}]^{2-}$ clusters	200	3	*16*

Supplementary Table 5. Summary of some recently reported representative HER electrocatalysts in alkali electrolytes (1.0 M KOH).

Catalysts	Catalyst loading amount (mg cm^{-2})	Overpotential (mV) at 10 mA cm^{-2}	Tafel slope (mV dec^{-1})	Ref.
**AC-Ru NSs**	81 μg_{Ru} cm^{-2}	**21**	**25**	**This work**
**A-Ru NSs**	81 μg_{Ru} cm^{-2}	**38**	**34**	**This work**
**C-Ru NSs**	81 μg_{Ru} cm^{-2}	**38**	**33**	**This work**
**AC-Rh NSs**	81 μg_{Ru} cm^{-2}	**32**	**28**	**This work**
**A-Rh NSs**	81 μg_{Ru} cm^{-2}	**37**	**36**	**This work**
**C-Rh NSs**	81 μg_{Ru} cm^{-2}	**38**	**40**	**This work**
**AC-Ir NSs**	81 μg_{Ir} cm^{-2}	**27**	**26**	**This work**
**Pt/C**	81μg_{Pt} cm^{-2}	**34**	**31**	**This work**
$RhSe_2$	336.82 μg cm^{-2}	82	96	*17*
RhPd-H	15.3 μg cm^{-2}	40	35.7	*18*
IrMo0.59	30 μg cm^{-2}	23	50	*19*
$Pt/Ni(HCO_3)_2$	204.1 μg cm^{-2}	27	45	*20*
IrP_2@NC	707.7 μg cm^{-2}	28	50	*21*
Ru@CN	246.8 μg cm^{-2}	32	53	*22*
Ru@GnP	250 μg cm^{-2}	22	28	*23*
Pt/MMC	69.4 μg cm^{-2}	45	30	*24*
Ni@Ni_2P-Ru HNRs	102 μg cm^{-2}	31	41	*25*

Supplementary references

1 Mahmood, J. *et al.* Encapsulating iridium nanoparticles inside a 3D cage‐like organic network as an efficient and durable catalyst for the hydrogen evolution reaction. *Adv. Mater.* **30**, 1805606 (2018).

2 Feng, J. *et al*. Iridium-based multimetallic porous hollow nanocrystals for efficient overall-water-splitting catalysis. *Adv. Mater.* **29**, 1703798 (2017).

3 Mahmood, J. *et al.* An efficient and pH-universal ruthenium-based catalyst for the hydrogen evolution reaction. *Nat. Nanotechnol.* **12**, 441-446 (2017).

4 Chao, T. *et al.* Atomically dispersed copper-platinum dual sites alloyed with palladium nanorings catalyze the hydrogen evolution reaction. *Angew. Chem.* **129**, 16263-16267 (2017).

5 Pu, Z. *et al.* RuP_2-based catalysts with platinum-like activity and higher durability for the hydrogen evolution reaction at all pH values. *Angew. Chem. Int. Ed.* **56**, 11559-11564 (2017).

6 Cheng, Y. *et al.* Rh-MoS_2 Nanocomposite Catalysts with Pt-Like Activity for Hydrogen Evolution Reaction. *Adv. Funct. Mater.* **27**, 1700359 (2017).

7 Jiang, P. *et al.* Pt-like electrocatalytic behavior of Ru-MoO_2 nanocomposites for the hydrogen evolution reaction. *J. Mater. Chem. A* **5**, 5475-5485 (2017).

8 Ying, J. *et al.* Nitrogen-doped hollow porous carbon polyhedrons embedded with highly dispersed Pt nanoparticles as a highly efficient and stable hydrogen evolution electrocatalyst. *Nano Energy* **40**, 88-94 (2017).

9 Zhu, L. *et al.* A rhodium/silicon co-electrocatalyst design concept to surpass platinum hydrogen evolution activity at high overpotentials. *Nat. Commun.* **7**, 12272 (2016).

10 Li, M. *et al.* Pt monolayer coating on complex network substrate with high catalytic activity for the hydrogen evolution reaction. *Sci Adv.* **1**, e1400268 (2015).

11 Huang, X. *et al.* Solution-phase epitaxial growth of noble metal nanostructures on dispersible single-layer molybdenum disulfide nanosheets. *Nat. Commun.* **4**, 1444 (2013).

12 Laursen, A. B. *et al.* Nanocrystalline Ni_5P_4: a hydrogen evolution electrocatalyst of exceptional efficiency in both alkaline and acidic media. *Energy Environ. Sci.* **8**, 1027-1034 (2015).

13 Jaramillo, T. F. *et al.* Identification of active edge sites for electrochemical H_2 evolution from MoS_2 nanocatalysts. *Science* **317**, 100-102 (2007).

14 Liang, H. W. *et al.* Molecular metal-Nx centres in porous carbon for electrocatalytic hydrogen evolution. *Nat. Commun.* **6**, 7992 (2015).

15 Popczun, E. J. *et al.* Highly Active Electrocatalysis of the Hydrogen Evolution Reaction by Cobalt Phosphide Nanoparticles. *Angew. Chem. Int. Ed.* **53**, 5427-5430 (2014).

16 Kibsgaard, J., Jaramillo, T. F., Besenbacher, F., Building an appropriate active-site motif into a hydrogen-evolution catalyst with thiomolybdate $[Mo_3S_{13}]^{2-}$ clusters. *Nat. Chem.* **6**, 248-253 (2014).

17 Zhong, W. *et al.* $RhSe_2$: a superior 3d electrocatalyst with multiple active facets for hydrogen evolution reaction in both acid and alkaline solutions. *Adv. Mater.* **33**, 2007894 (2021).

18 Fan, J. *et al.* Hydrogen stabilized RhPdH 2D bimetallene nanosheets for efficient alkaline hydrogen evolution. *J. Am. Chem. Soc.* **142**, 3645-3651 (2020).

19 Fu, L. *et al.* IrMo nanocatalysts for efficient alkaline hydrogen electrocatalysis. *ACS Catal.* **10**, 7322-7327 (2020).

20 Lao, M. *et al.* Platinum/nickel bicarbonate heterostructures towards accelerated hydrogen evolution under alkaline conditions. *Angew. Chem.* **131**, 5486-5491 (2019).
21 Pu, Z. *et al.* A universal synthesis strategy for P-rich noble metal diphosphide-based electrocatalysts for the hydrogen evolution reaction. *Energy Environ. Sci.* **12**, 952-957 (2019).
22 Wang, J. *et al.* Highly uniform Ru nanoparticles over N-doped carbon: pH and temperature-universal hydrogen release from water reduction. *Energy Environ. Sci.* **11**, 800-806 (2018).
23 Li, F. *et al.* Mechanochemically assisted synthesis of a Ru catalyst for hydrogen evolution with performance superior to Pt in both acidic and alkaline media. *Adv. Mater.* 3**0**, 1803676 (2018).
24 Baek, D. S. *et al.* Ordered mesoporous metastable α-MoC_{1-x} with enhanced water dissociation capability for boosting alkaline hydrogen evolution activity. *Adv. Funct. Mater.* **29**, 1901217 (2019).
25 Liu, Y. *et al.* Ru modulation effects in the synthesis of unique rod-like Ni@ Ni_2P-Ru heterostructures and their remarkable electrocatalytic hydrogen evolution performance. *J. Am. Chem. Soc.* **140**, 2731-2734 (2018).
